# Experimental research in environmentally induced hyperthermic older persons: A systematic quantitative literature review mapping the available evidence

**DOI:** 10.1080/23328940.2023.2242062

**Published:** 2023-08-27

**Authors:** Aaron J. E. Bach, Sarah J. K. Cunningham, Norman R. Morris, Zhiwei Xu, Shannon Rutherford, Sebastian Binnewies, Robert D. Meade

**Affiliations:** a School of Medicine and Dentistry, Griffith University, Gold Coast, QLD, Australia; b Cities Research Institute, Griffith University, Gold Coast, QLD, Australia; c School of Health Sciences and Social Work, Griffith University, Gold Coast, QLD, Australia; d Metro North Hospital and Health Service, The Prince Charles Hospital. Allied Health Research Collaborative, Brisbane, QLD, Australia; e Menzies Health Institute Queensland, Griffith University, Gold Coast, QLD, Australia; f School of Information and Communication Technology, Griffith University, Gold Coast, QLD, Australia; g Human and Environmental Physiology Research Unit, School of Human Kinetics, University of Ottawa, Ottawa, ON, Canada; h Harvard T.H. Chan School of Public Health, Harvard University, Boston, MA, USA

**Keywords:** Aging, chronic disease, climate change, heat exposure, heat strain, heat stress, heatwaves

## Abstract

The heat-related health burden is expected to persist and worsen in the coming years due to an aging global population and climate change. Defining the breadth and depth of our understanding of age-related changes in thermoregulation can identify underlying causes and strategies to protect vulnerable individuals from heat. We conducted the first systematic quantitative literature review to provide context to the historical experimental research of healthy older adults – compared to younger adults or unhealthy age matched cases – during exogenous heat strain, focusing on factors that influence thermoregulatory function (e.g. co-morbidities). We identified 4,455 articles, with 147 meeting eligibility criteria. Most studies were conducted in the US (39%), Canada (29%), or Japan (12%), with 71% of the 3,411 participants being male. About 71% of the studies compared younger and older adults, while 34% compared two groups of older adults with and without factors influencing thermoregulation. Key factors included age combined with another factor (23%), underlying biological mechanisms (18%), age independently (15%), influencing health conditions (15%), adaptation potential (12%), environmental conditions (9%), and therapeutic/pharmacological interventions (7%). Our results suggest that controlled experimental research should focus on the age-related changes in thermoregulation in the very old, females, those with overlooked chronic heat-sensitive health conditions (e.g. pulmonary, renal, mental disorders), the impact of multimorbidity, prolonged and cumulative effects of extreme heat, evidence-based policy of control measures (e.g. personal cooling strategies), pharmaceutical interactions, and interventions stimulating protective physiological adaptation. These controlled studies will inform the directions and use of limited resources in ecologically valid fieldwork studies.

## Introduction

A globally aging population [1], growing rates of noncommunicable diseases (e.g. cardiovascular disease) [2], and a warming climate [3] will see older people continue to carry a disproportionate burden of excess illnesses and deaths from extreme heat [4,5]. It is well established that older adults are at elevated risk of adverse heat-related health events due to age-related declines in the physiological systems tasked with maintaining homeostasis during heat stress. The etiology is multifactorial and has been highlighted in numerous narrative reviews [6–12]. As we age, several key biological pathways are impacted compared to healthy younger counterparts, lessening our body’s ability to remove excess heat and maintain systemic perfusion. These alterations include changes to endothelial function [13], autonomic vascular responses [14], and cardiac sufficiency [15], which impact hemodynamic regulation; reductions in sweat production per unit change in body temperature – impacting the body’s evaporative cooling capacity [16] and impaired regulation of fluid balance – impacting renal water retention [17], sensations of thirst [17,18], and fluid-saving adjustments to sweat rate during dehydration [19,20]. Together, these alterations mean that older adults are subjected to higher levels of hyperthermia and associated physiological strain during heat exposure, even under compensable conditions [6].

While aging is perhaps the single most influential non-modifiable risk factor of heat-vulnerability, there are several factors known to modify thermoregulatory function and the risk of heat-related illness and injury in older adults [21–23]. For example, females have a reduced capacity to dissipate heat [24] and are at greater risk of heat-related mortality during hot weather [25,26]. Further, future projections of global disease burden indicate marked increases in diseases known to increases the risk of heat-related morbidity and mortality such as heart failure [27] and type 2 diabetes (T2D) [28]. Often chronic disease states are not suffered in isolation. For example, the underlying cause of chronic kidney disease is often hypertension and/or T2D [29]. In the United States, over 60% of adults aged 65 or older suffer from two (co-) or more (multi-)chronic diseases [30]. Co- and multi-morbidities can compound heat health risk and are expected to be more prevalent amongst the world’s aging population [31–33]. Further complicating these scenarios are the associated medications (e.g. anticholinergics, vasodilators, diuretics, negative chronotropics) or treatments (e.g. fluid restriction) that can further dampen the physiological capacity to respond to elevated ambient temperatures [34,35].

While much primary experimental research to understand aging and thermophysiology during heat exposure has been carried out since the mid-20^th^ century, none have systematically quantified the volume of evidence, associated the individual factors of interest, or how secondary factor interaction may modify heat risk. More experimental studies on the effects of age-related changes in thermoregulation are needed to further understand the putative mechanisms contributing to increased risk of heat-related mortality, but also point toward effective strategies for protecting older adults (e.g. physical training, acclimatization, personal cooling, pharmacological interventions, etc.). However, these studies entail considerable practical challenges due to their expense, complex and costly equipment, time commitment, and reliance upon exposing a vulnerable group to extreme heat. As such, environmental physiologists must be sure to address the “*big questions*” with the limited resources at hand, and for that, there is a need for a systematic review of what has been done and what is still needed to better shape future research directions.

Systematic quantitative literature reviews (SQLR) are a relatively new technique that contrasts from meta-analysis or subject-specific systematic reviews by quantitatively mapping topic boundaries and identifying trends within a discipline [36,37]. Here, for the first time, we aim to provide broader context to the experimental research of healthy older adults (≥50 years) – compared to younger adults or unhealthy age matched cases – during exogenous heat strain, focusing on factors that influence thermoregulatory function (e.g. fitness, co-morbidities, medications). This review sets out to identify historical trends, current knowledge gaps, and potential avenues for future experimental research and/or meta-analyses. Specific attention is paid to known health conditions that influence heat health risk and the ability of current evidence to improve our understanding of heat health risk of older persons during heatwaves in a warming world.

## Methodology

The databases PubMed, Scopus, CINAHL, Web of Science, and SportDiscus were searched from database inception up to June 2023. The search was limited to original research articles published in English but not publication date. Eligibility criteria are presented in Table 1. The age cutoff of studies with a group mean of ≥50 years was selected as it is the common lower bound for middle-older age in experimental physiology research, with decrements in heat loss capacity observed in healthy persons as young as 40 years of age [38,39].

**Table 1.** Eligibility criteria were developed to target peer reviewed publications of primary experimental research.

Inclusion criteria	Exclusion criteria
Design must be RCT/experimental, quasi-experimental, cohort study, or other suitable analytical design	Reviews, meta-analyses, incomplete clinical trials, instrumentation studies, editorials, commentary, letters, news articles
The case group must have a mean age of at least 50 years or older	Studies reporting on heat shock proteins as their only outcome of interest
Case group(s) must either be same age or health and risk factor status as control group	Anaesthesia setting where effect of anaesthetic on thermoregulation is outcome of interest
Must report participant thermoregulatory response measures (e.g., heat loss, core or skin temperatures, skin blood flow)	Any study where heat is incidental or a result of the primary therapy investigated (e.g., vibrational or localised applied heat for wound management, chemotherapy, dialysis, nociception, etc.)
Exposure must elicit whole body heating in a hot environment (air or water including water perfused suits) via passive (i.e., rest) and/or active (i.e., exercise) means	Animal, cadaveric, ex-vivo, in-vitro, or hypothetical and modelling studies

A PICO-style method (*Population* = adults ≥50 years; *Intervention* = exogenous heat exposure; *Comparison* = adults <50 years or unhealthy adults ≥50 years; and *Outcomes* = thermoregulatory function) was employed to help draft keywords [40]. An initial search was conducted in August 2022 to check for result suitability. From this initial search, a sample of 10 relevant source papers were examined through “Word Freq” tool within the Systematic Review Accelerator [41] to identify any missed keywords or synonyms. The Boolean search string was able to return the 10 relevant source papers in “*SearchRefinery*” tool within the Systematic Review Accelerator [41] to determine which PICO elements were yielding the most relevant results. This combined with librarian feedback demonstrated that terms relating to comparison and outcomes measures were either redundant or inadvertently excluding some records. Therefore, the search was simplified to terms relating to age and heat exposure or thermoregulation, with removed elements to be applied via screening. After building the Boolean string in PubMed, the “Ployglot Search” tool within the Systematic Review Accelerator [41] was used to assist in translating the searches to other database query formats. The final search strings for each database are presented in Table S1 of the accompanying *Supplement*.

Screening of titles, abstracts, and full texts, as well as data extraction was performed by two of three authors (SC, ZX, AB), with one author consistent throughout screening (AB). Disagreements were handled by consensus, with an independent author (SR) enlisted if a consensus could not be reached. From the included studies, one author (AB) conducted a backwards citation screen via the “SpiderCite” tool within the Systematic Review Accelerator [41] to find other sources outside of the original search. The systematic review and data extraction was carried out following the methods outlined in Pickering and Byrne (2014) [36] and Pickering et al. (2015) [37]. An essential component of the SLQR method is to categorically organize literature as a means of better understanding trends and gaps in the field. Data extracted from the papers included general information (bibliometrics, study design, objectives, and findings), study categorization (adaptation, condition, biological mechanism, environment, pharmacological), methodological qualities (sample characteristics, environmental parameters), and thermal physiological measures (primary and secondary measurements) (Table 2).

**Table 2:** Categories of data and table of definitions

Element	Description of categories		Data type
Bibliometrics	*Authors, Country, Institution, Year, Title, Journal*		Descriptive
Objectives and findings	Summary of key objective and findings of report		Descriptive
Design	Reports were categorised by their relevant experimental comparison groups, of either *Younger v Older* where age is the independent variable or *Older v Older* where another factor was the independent variable. Where one report had more than one relevant experimental comparison group, each comparison was extracted as its own study. Irrelevant experimental groups were ignored (e.g., paediatric groups).		Categorical
Factor categories and subcategories	*Biological mechanism* A cellular pathway involved in heat thermoregulation	*NOS-dependent pathway, Orthostatic tolerance, Biomarkers, Regional sweat, Antioxidant activity, Cardiovascular activity, Nervous activity, Renal function*	Categorical
	*Condition* A medical condition or physical characteristic that may affect thermoregulation	*Breast cancer, Diabetes, Heart failure, Hypertension, Ischemic heart disease, Multiple system atrophy*	
	*Adaptation* A heat adaptation mechanism or strategy	*Fitness, Acclimation, Hydration, Work intensity, Seasonal, Cooling intervention*	
	*Environment* A difference in environmental exposure	*Temperature, Humidity, Air velocity*	
	*Therapeutic/pharmacological* A drug or hormone that may affect heat thermoregulation	*Aspirin, Folic acid, Hormone replacement therapies, Clopidogrel, Thiazolidinedione, Rosiglitazone, Sapropterin*	
	*Other*	*Biological sex, occupation, clothing*	
Sample characteristics	Demographics	*Total Number of Participants, Number of Males, Number of Females, Number of Controls, Number of Cases, Total and Group Ages [Mean, Min, Max].*	Integer
	Baseline data Fitness	*VO_2_ [Peak/Max/Estimated], Heart rate [Peak/Max], Health or Fitness screening*	Binary (Reported/Absent)
	Physical characteristics	*Height, Mass, Body surface area, Body fat, Blood pressure*	
	Other baselines measures	*Medication, Vascular disease, Respiratory disease, Cognitive or Neuropsychiatric disease, Other comorbidities*	
Thermophysiological measures	Body temperature	*Skin, Rectal, Oesophageal, Sublingual, Gastrointestinal, Tympanic, Cardiopulmonary, Muscular, Not stated*	Binary (Reported/Absent)
	Cardiovascular	*Heart rate, Stroke volume or Cardiac output, Respiration rate, Mean arterial pressure or Systolic/Diastolic blood pressure, Skin blood flow or Forearm blood flow or Cutaneous conductance, Plasma volume*	
	Fluid and heat balance	*Urine specific gravity, Urine osmolality, Sweat rate*	
	Heat and work	*Heat balance, Relative perceived exertion, Thermal comfort*	
	Other	*Cognitive performance, Psychological wellbeing, Blood assay, Physical performance, Other measures*	
Exposure procedures	Exercise	Method *Cycling, Walking, Running, Isometric*	Categorical
		Length *Per session minutes, Reported or calculated mean sessions minutes, Total exercise time minutes*	Continuous
		*Number of sessions*	Integer
	Heat exposure	Length *Total heat exposure minutes, Reported or calculated mean total heat exposure minutes including rest*	Continuous
		Method *Chamber, Water immersion, Perfused suit*	Categorical
		Number of sessions	Integer
		Temperatures *Control temperature, Overall experimental temperature mean, Overall experimental dry-bulb temperature mean, Overall experimental-bulb temperature mean, Overall humidity mean, Water immersion temperature mean, Perfused suit temperature mean, Minimum experimental temperature, Maximum experimental temperature*	Continuous

Data manipulation and analyses were conducted in R (version 4.2.1) [42], using the RStudio environment (version 2022.07.1 Build 554) [43] with data visualizations produced using the R package “*ggplot2*” [44].

## Results

**Figure 1.**
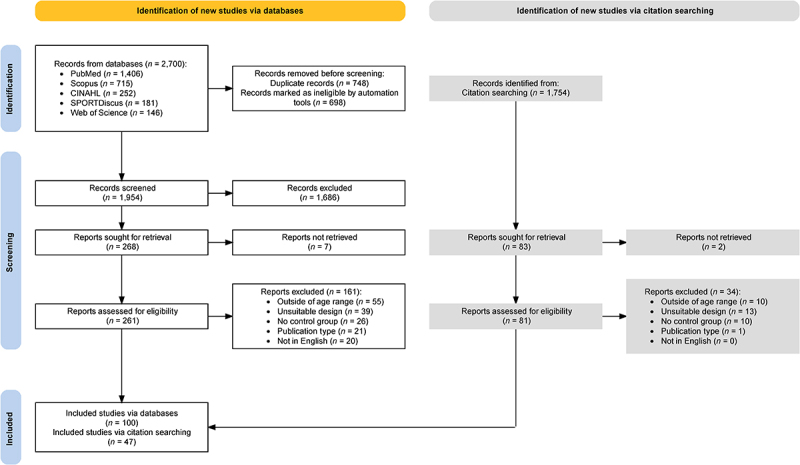
PRISMA flow diagram of included studies [45,46].

A total of 4,455 articles were returned and screened leading to a total of 147 studies included in the review (Figure 1). At least 16 of the 147 studies appeared to be directly linked to another included publication, but it was not always clear if any or what proportion were the same participants, therefore weightings were not applied to balance extracted data.

As this review was concerned with both the effect of age, and factors in the aged, the proportion of studies that examined each was of primary interest. Some studies had more than two participant groups and considered both age and other factors (e.g. health status), or multiple other factors (e.g. sex, health status). Where a single study had multiple relevant participant groups, each comparison of groups was counted separately giving a total of 167 unique between/within group comparisons relating to age (e.g. young vs older) or age-related factors (e.g. older healthy vs older unhealthy).

**Figure 2.**
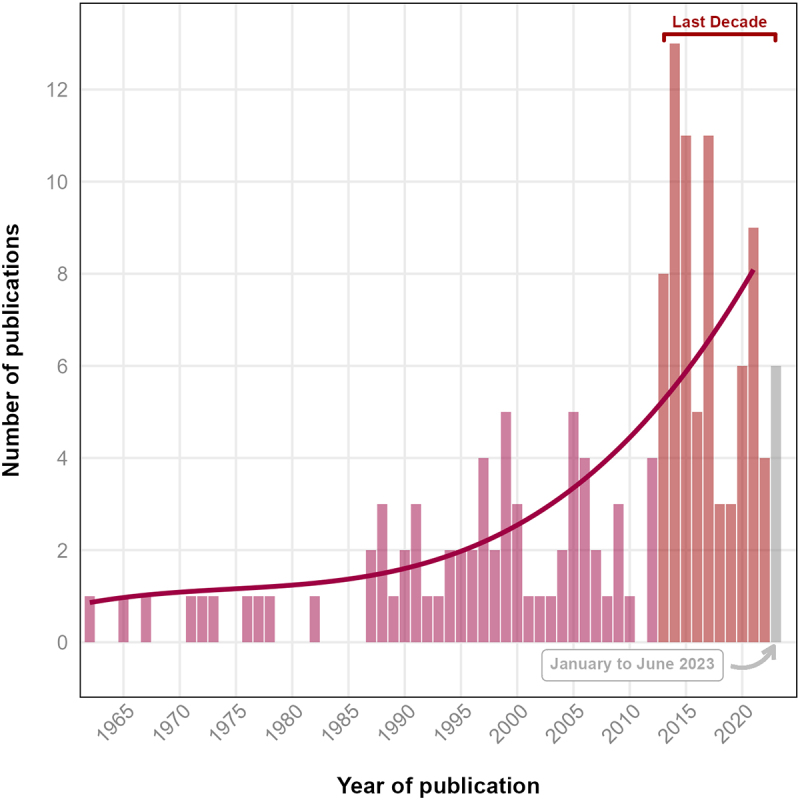
Temporal trend of experimental studies in environmentally induced hyperthermic older persons.

**Figure 3.**
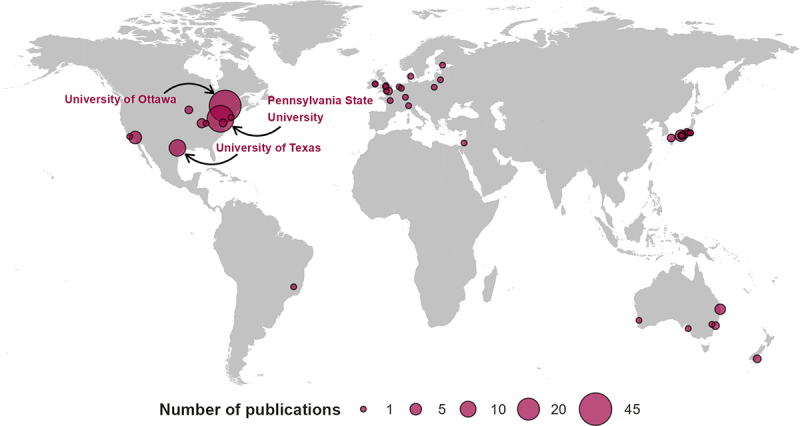
Location and frequency of included studies by the leading institution.

The included studies (*k =* 147) spanned publication dates between 1962 and 2023 (Figure 2). There is an accelerating publication rate of controlled experimental trials studying independent risk factors for heat stress in older adults with 79 (53%) studies published in the last decade. Countries publishing works were United States (*k =* 57, 39%), Canada (*k =* 43, 29%), Japan (*k =* 18, 12%), Australia (*k =* 9, 6%), UK (*k =* 5, 3%), NZ (*k =* 2, 1%), Ireland (*k =* 2, 1%), France (*k =* 2, 1%), Netherlands (*k =* 2, 1%), and a single paper each from Brazil, Denmark, Finland, Israel, Italy Lithuania, and Poland (Figure 3). Fifty-seven percent (*k* = 84) of studies were led by just three institutions; the University of Ottawa (*k* = 43), Pennsylvania State University (*k* = 30), and University of Texas (*k* = 11).

**Figure 4.**
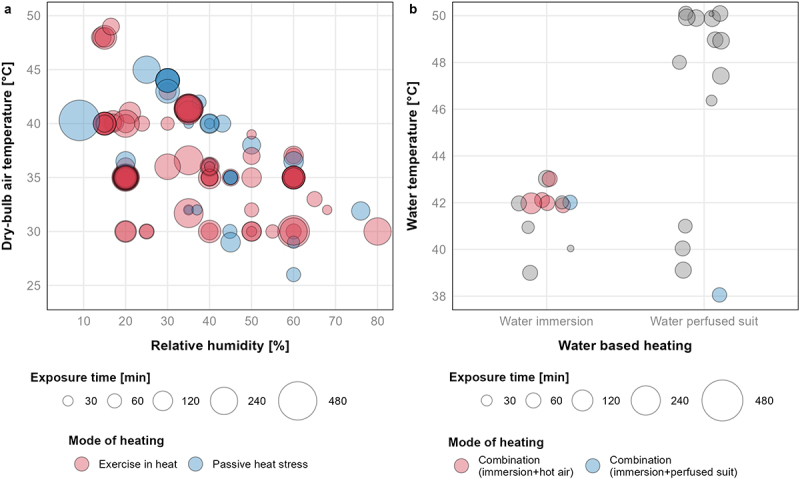
Magnitude, duration, and means of heat exposure; a) air-based exposures, and b) water-based exposures. Visualisation of plot a 107 conditions from 95 studies. Visualisation of plot B includes 31 conditions from 27 studies. Twelve additional studies were excluded from plot A; one study for visualization purposes (85°C; 3.5% *ϕ*; 10 min exposure), three studies did not report relative humidity, seven studies did not report mean conditions and/or exposure times due to inherent variability in testing methodology, and one study only reported a range of wet-bulb globe temperature (26–29°C). Thirteen additional studies were excluded from plot B; 12 studies did not report mean exposure lengths due to core temperature clamp protocols (water temperatures: *k* = 1 at 38°C, *k* = 1 at 41°C, *k* = 3 at 46°C, *k* = 2 at 48°C, *k* = 3 at 50°C, *k* = 2 at 52°C), and one study did not report a mean water perfused suit temperature. Where multiple age groups were defined, visualizations only include the oldest and youngest comparisons.

From a total of 130 studies that reported mean heat exposure lengths, the median exposure duration was 100 min (interquartile range [IQR]: 60–165 min). In 75 studies, physical exertion combined with high ambient temperatures was used to illicit heat stress (Table 3; Figure 4(a)). The remaining 73 studies prescribed a single passive method or combined method of passive heat stress (37 exposing participants to elevated air temperatures, 29 using a water-perfused suit, and/or 13 using water immersion) (Table 3; Figure 4(b)).

**Table 3.** Summary of 148 included study characteristics.

Element		Result	n	%
Study sample		*Young v Older Comparisons*	103	^*a*^
		*Older v Older Comparisons*	53	^*a*^
Study characteristics				
	Screening	*Medical/fitness screening*	122	83
		*Medication screening*	99	67
	Baseline characteristics	*Height*	124	84
		*Mass*	136	93
		*Body surface area*	89	61
		*Body fat*	83	56
		*Blood pressure*	44	30
		*VO*_*2*_*(peak or max)*^*b*^	81	55
		*Heart rate (peak or max)*^*b*^	25	17
Exposure protocols				
	Mode of heating	*Environmental chamber*	100	68
		*Perfused suit*	28	20
		*Water immersion*	5	3
		*Water immersion + chamber*	7	5
		*Water immersion + perfused suit*	1	<1
		*Not stated*	6	4
	Modality	*Resting (passive) heat stress*	74	50
		*Exertional (active) heat stress*	73	50
	Duration (single exposure)	*<30 min*	14	9
		*31-60 min*	21	14
		*61-120 min*	46	31
		*121-180 min*	34	23
		*181-240 min*	10	7
		*>240 min*	6	4
		*Variable/not stated*	16	11
	Environmental reporting	*Temperature (NFD)*	109	74
		*Dry bulb temperature*	16	11
		*Wet bulb globe temperature*	18	12
		*Relative humidity*	107	72
		*Air velocity*	31	21
		*Water temperature*	41	28
	Exercise application	*No exercise*	73	50
		*Walking/running*	16	11
		*Cycling*	57	39
		*Isometric*	1	<1

Note: ^*a*^ = as some studies had multiple comparisons (e.g. older healthy, older unhealthy and younger healthy groups) the number of total comparisons counted exceeds the 147 included studies. ^*b*^ = measured or estimated. NFD = not further defined (presumably dry bulb/air temperature).

**Figure 5.**
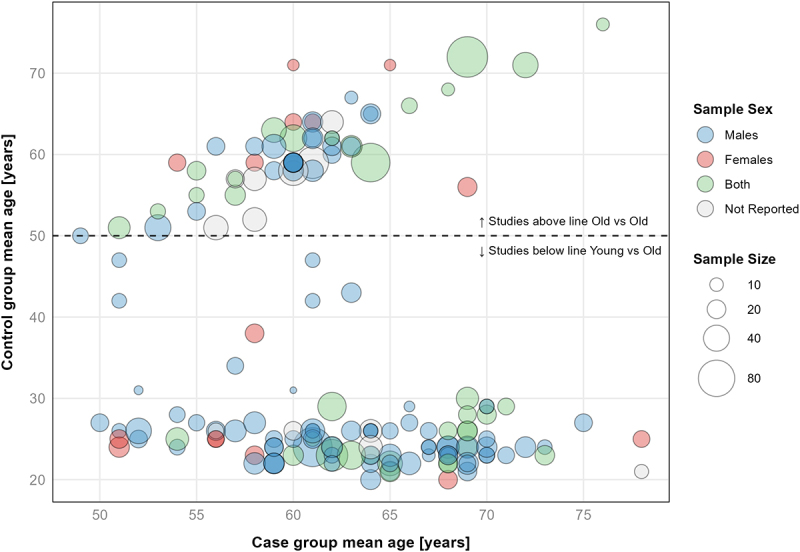
Sample size, sex, and mean age of groups in included studies. Visualisation includes 137 studies, with 161 paired comparison groups. Eleven studies, with a total sample size of *n* = 336, did not report mean age of their study groups. As per screening criteria older was defined as ≥50 years.

The median number of total participants within each study was *n* = 20 (IQR: *n* = 16–25), for control groups *n* = 10 (IQR: *n* = 8–14), and for case groups *n* = 10 (IQR: *n* = 8–14). A total number of 3,411 adults participated in the studies of whom 2,428 (71%) were males, 647 were (19%) females, and 336 (10%) participants’ sex was not reported. The reported mean age of the case groups (i.e. the older group(s) within an included study) were distributed across the following binned age ranges, 50-54y: *n* = 16, 55-59y: *n* = 32, 60-64y: *n* = 59, 65-69y: *n* = 36, 70-74y: *n* = 13, ≥75y: *n* = 4. The mean age, average sample size, and sex distribution of screened studies are presented in Figure 5.

**Figure 6.**
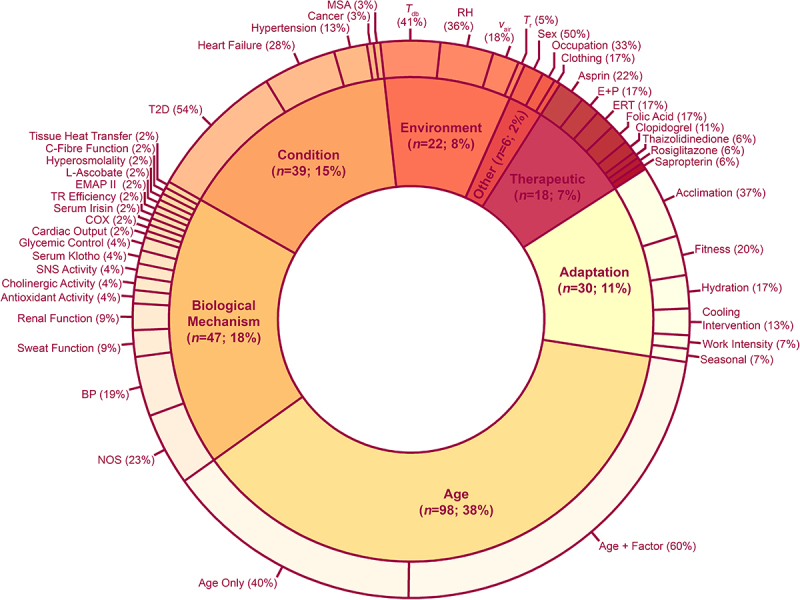
Factor categories and subcategories within included studies. Many studies had multiple independent factors of interest, leading to 260 comparisons within 147 studies. BP = blood pressure; COX = cyclooxygenase; E+P = estrogen and progesterone; EMAP II = endothelial monocyte‐activating polypeptide‐II; ERT = estrogen replacement therapy; MSA = multiple system atrophy; NOS = nitric oxide synthase; RH = relative humidity; SNS = sympathetic nervous system; T2D = type 2 diabetes; *T*_db_ = dry bulb air temperature; *T*_r_ = radiant temperature; TR = thermoregulatory; *v*_air_ = air velocity.

Most studies (*k* = 103) made comparisons between younger and older adults, with 53 studies making comparisons between two groups of older adults with and without a factor(s) known or suspected to influence thermoregulation (Figure 6). Of these, nine studies were able to make both old (factor) vs old (non-factor) and old vs young comparisons. Key factors for thermoregulatory function included studies focusing on effect of age with another factor (*k* = 60, 23%) underlying biological mechanism(s) (*k* = 47, 18%), age independently (*k* = 38, 15%), influencing health conditions (*k* = 39, 15%), adaptation potential (*k* = 30, 12%), different environmental conditions (*k* = 22, 9%), therapeutic interventions (*k* = 18, 7%) (Figure 6). The thermophysiological variables measured in the included studies are tallied and presented in Table 4.

**Table 4.** Summary of thermophysiological variables measured in the 147 included studies.

Measure	Variables	No. studies (*k*)	%
Temperature			
	*Skin*	113	77
	*Rectal*	71	48
	*Oesophageal*	33	22
	*Sublingual*	17	12
	*Gastrointestinal*	18	12
	*Tympanic*	5	3
	*Cardiopulmonary*	1	<1
	*Muscular*	1	<1
	*Not Stated*	1	<1
Cardiovascular			
	*Heart rate*	115	78
	*Stroke volume, cardiac output*	24	16
	*Respiration rate*	6	4
	*Blood pressure (MAP/SD)*	77	52
	*Plasma volume*	17	11
	*SkBf/CVC/FBF*	73	50
Fluid balance			
	*Sweat rate*	68	46
	*Urine specific gravity*	17	12
	*Urine osmolality*	2	1
Heat and work			
	*Heat production/heat balance*	34	23
	*Thermal comfort*	17	12
	*Perceived exertion*	11	7
Other measures			
	*Blood assay*	21	14
	*Physical performance*	11	7
	*Cognitive performance*	3	2
	*Psychological wellbeing*	1	<1

Note: MAP = mean arterial pressure; SD = systolic/diastolic; SkBf = skin blood flow; CVC = cutaneous vascular conductance; FBF = forearm blood flow.

## Discussion

In this systematic quantitative literature review, we compiled the independent factors previously investigated in experimental trials focused on thermoregulation in older adults during heat exposure. Experimentally controlled studies on the impact of T2D and heart failure were the most investigated chronic health conditions. However, studies to date have largely ignored the sex-based differences, those over 75 years, prolonged and compensable heat stress, cooling strategies for older adults, and other heat-sensitive diseases. To aid in the development of future work, the following sections detail the current limitations and knowledge gaps in this field, with particular attention paid to demographic sampling, experimental design, and ecological validity for heatwaves, and the impact of heat-susceptible disease states.

### Demographic considerations

Age is the strongest non-modifiable predictor of heat-vulnerability [47,48]. Epidemiological evidence suggests that during heatwaves, persons 65 years or older are at higher risk of heat-related mortality than their younger counterparts, with risk potentially rising in even older age groups (i.e. the oldest-old) [49,50]. Despite this, only four (3%) of the 147 included studies reported a mean age of their older sample to be 75 years or more. Given the elevated risk experienced by these individuals, particularly those with age-associated chronic health conditions (discussed below), their inclusion in future work is important to bettering our understanding of age-related declines in thermophysiological function and their putative links to heat vulnerability. Where possible, further experimental studies in persons aged 75 years and older will better inform (the most likely non-linear) risk profiles in the most vulnerable age groups.

Of course, extending the upper age range of thermophysiolgoical studies carries key ethical and logistical considerations. The study eligibility criteria should be carefully formulated to avoid recruiting participants at undue risk of heat-related health events. Detailed health and medical screening, which can include physical fitness evaluation and cardiac stress testing, and physician clearance should be used to mitigate participant risk. While stringent screening of participant should improve safety when working with potentially vulnerable groups, it also carries the added benefit of minimizing the influence of confounding factors (e.g. cardiorespiratory fitness, history of heat exposure), improving internal validity. It should be noted, however, that while these constraints will improve participant safety and allow for greater understanding of age and/or disease-related thermoregulatory degradation in the very old, they can lead to homogenic groups not necessarily reflective of the most vulnerable during hot weather and heatwaves. Extending experimental physiological research to evaluate responses in the oldest-old while ensuring participant safety and internal validity without sacrificing ecological relevance represents a key challenge to improving our understanding of the physiological basis of age-related heat vulnerability and how it can be mitigated.

### Key modifiers of the relation between age and thermoregulation function

#### Common age-associated chronic health conditions

It is well established that common age-associated chronic health conditions increase the risk of heat-related mortality and morbidity and many of these conditions have also been shown to exacerbate age-related declines in thermoregulatory function 6,47,]. Of the 32 studies investigating the modifying effect of chronic diseases on age-associated declines physiological responses to heat stress, T2D was the most common (*k* = 21), although one study investigated both Type 1 and Type 2 as a single diabetic comparison group [51]. Heart failure was the second most investigated condition (*k* = 10). T2D and heart failure seem to be likely candidates in independent systematic reviews, though the heterogeneity in study methodology and participant characteristics would need to be explored to determine if formal meta-analysis is feasible.

The focus of included studies with diabetic comparison groups was typically investigating an underlying mechanism or sequalae of T2D that was responsible for possible maladaptations to increased heat stress. Impaired vascularity and epithelial function are known complications of T2D [52], consequently this is an area of interest in heat adaptation. When exposed to increasing temperatures, both older and T2D groups reached a maximum threshold of increased skin blood flow earlier than younger healthy controls [53]. Similarly, those with T2D have been found to have a higher core body temperature onset, or delayed vasodilation response, compared to healthy older people [54] and have demonstrated a compromised sudomotor function [55]. Consequently, older adults with T2D have been shown to exhibit reduced heat loss and greater heat storage compared to age-matched counterparts without the disease in exercise-based studies approaching or exceeding the upper limits of compensability, resulting in considerably less heat loss and greater heat storage in those older adults with T2D [10,56]. Interestingly, ensuing work found no differences in whole-body heat loss, heat storage, or body temperatures between older adults with and without T2D resting for 3 hours in environmental heat stress (43°C and 35% RH) [57].

While studies on T2D focused primarily on the thermoregulatory consequences of the disease, identified studies evaluating outcomes in persons with heart failure generally focused on the cardiovascular and fluid regulatory outcomes. This is perhaps unsurprising since heart failure is characterized by decreased cardiac function and altered autonomic function, which impairs blood flow regulation needed to cope with heat stress [58]. Compounding this, diuretic use, a common method for treating heart failure has been linked to decreased skin blood flow due to reduced plasma volume [59], which might further impair thermoregulatory function. The delicate fluid balance required in treatment of heart failure can place patients at risk of heat-related illness if they become dehydrated as a consequence of water intake restriction [60]. Previous exercise and passive heat-based studies of heart failure patients have pointed to reduced skin blood flow, via attenuated vasodilatory control and cardiac reserve, as the primary thermoregulatory maladaptation [61–63]. However, these studies included participants who were still on standard therapy (i.e. prescribed beta-blockers) [64]. Though it is likely that attenuated skin blood flow (of up to 20%) is largely inconsequential to overall thermal strain when ambient temperatures are extremely high (>40°C) [65,66]. Notwithstanding, it is critical to further understand the extent of greater heat vulnerability in older persons with heart failure, along with the interaction of commonly associated medications.

Multimorbidity (≥2 health conditions) is prevalent in the elderly population [67]. In addition to T2D and heart failure, epidemiological evidence suggests that underlying cardiovascular diseases (e.g. hyperlipidemia [68,69], hypertension [47]), mental disorders (e.g. dementia) [70,71], respiratory conditions [47], renal dysfunction [72], and are associated with elevated risk of heat-related morbidity and mortality. It is currently unclear, however, the extent to which disease-related alterations in physiological responses to heat exposure contribute to this increased risk both in isolation and when combined with other conditions. For example, while individuals with chronic obstructive pulmonary disease (COPD), may be at higher risk of complications during hot weather [47], more research is needed as to whether this is due to a reduced physiological capacity for temperature regulation, the circumstances by which COPD is often a multimorbid condition, or due to reductions in air quality associated with hot weather [73].

Future work should explore thermophysiological function of other chronic diseases which are yet to be evaluated experimentally in the heat (e.g. high-grade hypertension, respiratory diseases, dementia, and kidney diseases). Anecdotally, while multiple sclerosis was well-represented publications that were part of review screening, all were ineligible for this review as they did not investigate any effect of age during heat exposure in their experimental design. As chronic diseases rarely occur in isolation, future more complex research would also, ideally, work to develop a greater understanding of the synergistic and antagonistic interactions of multimorbidity and accompanying medications pose on heat-health risk in older adults during simulated or lived heatwaves. Caution is warranted, however, as confidently evaluating interactive effects will require careful yet extensive study given the large number of heat-vulnerability-linked diseases and medications [74,75].

#### Sex

Within the wider field of thermophysiology research, pre-menopausal women are often overlooked due to perceived difficulties in controlling for hormonal and contraceptive-based influences on thermoregulation [76]. This trend seems to hold true for studies of older adults included in this review, with older women comprising only 19% of total participants. Sex differences have been shown in older adults, with older women demonstrating a lower whole-body heat loss during moderate-to-vigorous exercise in a dry heat environment compared to age-matched males [24]. Though it is not clear if these responses are present during prolonged heat exposures where the upper limits of compensable heat stress are not often exceeded (i.e. passive hot weather conditions). Regardless, given that there are known sex differences in the risk of heat-related mortality [25,26] and heat loss capacity in older adults during exertional heat stress [24], along with the common demographic proportions in aging (i.e. women on average living longer and making up a larger percentage of older persons) [1] more research is needed to better understand if there are meaningful changes between sexes with aging, and to what extent sex-specific therapeutics (such as exogenous hormones for menopause) influence heat-health risk.

### Toward ecologically relevant study designs

Most studies included in this review induced heat stress via exercise in the heat, encapsulation via hot water-perfused suits, or hot water immersion (*k* = 114, 78%). These modalities have the major advantage that heating is achieved rapidly, which facilitates comparisons between different groups (e.g. young, and older adults). Whilst these studies are critical in better understanding the potential deficits in heat loss capacity in older adults, their reflectiveness of the heat stress experienced in real-world conditions such as during hot weather or heatwaves has been questioned [6]. In this review, only eight (5%) included studies set out to specifically replicate heatwave conditions (i.e. passive exposures ≥180 min) [57,77–83]. Regardless, the studies included in this review tended to prescribe conditions more extreme than those likely experienced by the most vulnerable persons during heat waves (e.g. most employed conditions similar to peak outdoor conditions; discussed further below). They were also conducted at a fixed temperature and relative humidity, over a shorter duration, employed a single exposure thus ignoring cumulative effects of heat exposure, focussed on waking hours with no emphasis on the primary and secondary effects of elevated overnight temperatures on heat recovery/sleep and subsequent heat-health risk, and often manipulated only air temperature in the absence of different contributions from radiant heat loads, air velocities, and/or water vapor pressures. Only 21 (14%) included studies manipulated multiple ambient parameters (e.g. temperature, humidity, airspeed, thermal radiation) between groups to understand their contributions to thermoregulatory strain.

Older adults spend more time at home than other age groups [84] and are estimated to spend up to 90% of their time indoors [85]. Although outdoor temperatures during heat events have a strong correlation with greater ambulance calls, emergency room presentations, hospital admissions, and mortality [86], the site of exposure – typically indoors at home – can vary significantly from those outdoors. This can be due to neighborhood and dwelling level factors (e.g. paved surfaces, vegetation) and individual behavior (e.g. uses of cooling strategies) [87,88]. Heat exposure characteristics unique to indoor settings may be more predictive of risk than more accessible outdoor parameters. Whether that be as a result of discrepancies between indoor-outdoor temperature and relative humidity [49,89–91], the observed cumulative and divergent rise in indoor temperatures over successive heat days [92], or the overnight indoor conditions being markedly higher than those outside [93].

To understand the risk of heat-related injury and illness in older persons, it is important to consider these effects of prolonged exposure to compensable heat stress elevating core temperature. This can occur over several days during heatwaves. Older males have shown a reduction in total heat loss resulting in greater whole-body heat storage following consecutive days of simulated work in the heat [94]. At a population level, air temperature thresholds for cardiovascular mortality have been shown to occur at lower average outdoor air temperatures (27°C vs 30°C) when heat events last three days rather than a single day [95]. Future work is needed in quantifying to what extent, if any, consecutive days of prolonged, passive indoor heat exposure act as a potential force multiplier for risk by reducing older persons’ heat loss capacity and subsequently increasing body heat storage.

### Protective strategies and future opportunities

Ultimately, a better understanding of age-related alterations in thermoregulatory function would lead to improved research on effective strategies for protecting the health and wellbeing of older adults during heat waves. Research is needed in evaluating practical strategies for mitigating heat stress in different environments, health conditions, and age groups. While air-conditioning is the strongest protective strategy for heat-related mortality [96,97], its growing reliance across the globe [98,99] is in direct conflict with the need to curb carbon emissions to mitigate anthropogenic climate change. Even where air conditioning is available to vulnerable older adults during heat events, concerns about energy costs [100,101] or impaired age-related thermal perception [102] may prevent its effective operation. Sustainable and practical alternatives than can facilitate human heat transfer to the environment are necessary. To date, almost all sustainable cooling strategies applied at the individual level have been studied in young adults, with few exceptions for older adults [103–106]. One example is that of electric fans, which have been shown to exacerbate hyperthermia in older, but not in younger men, in response to rising ambient humidity during passive exposure to extreme heat (42°C) [104]. These responses are most likely due to an increase in dry convective heat gain, the negative effects of which are diminished by a superior sweating response in younger participants relative to older adults. The differences are likely further augmented when people are taking specific medications (e.g. anticholinergics) [107]. Hence, building upon the current evidence-base for quantifying the magnitude of change anticholinergics, amongst other medications, have on specific mechanisms of thermoregulation (e.g. skin blood flow, sweat output, thermal sensation) is an additional area of ongoing need.

In addition to individual level cooling interventions, more experimental research is required to evaluate the efficacy of methods for improving personal heat resilience. A recent systematic review of short-term exercise-based heat acclimation in older adults (50–70 years) concluded it to be an effective method at producing positive adaptations for heat loss capacity and exercise performance in the heat [108]. It is less clear if short-term exercise-based heat acclimation provides the individual ample protection during prolonged and compensable heat events, and if it can be prescribed for the most vulnerable less able to exercise (i.e. those with co-morbidities, mobility concerns, and/or the very old). Though the use of hot water immersion therapy seems to confer partial physiological adaptations when supplemented with low-intensity exercise [109]. More research is needed to determine the independent protective effects of acclimation via hot water immersion when applied to those unable to exercise. Controlled experiments for evaluating personal cooling strategies, pharmaceutical interactions, or physiological adaptations can be the first stage of a two-step process of data collection, whereby climate chamber studies identify from a range of options, promising and pragmatic solutions to mitigate heat-health risks. The ongoing work can be focused and upscaled to field-based experiments to see if efficacy of a given strategy in a controlled laboratory environment translates to effectiveness in the real world.

## Summary

In summary, we present the first systematic quantitative literature review to better define the current research landscape of age-related decrements in thermoregulation in adults over 50 years old. This systematic review focused on controlled experimental research in older adults exposed to heat. In the past six decades, there is an accelerating publication rate of controlled experimental trials studying independent risk factors for heat stress in older adults. The studies included in this systematic quantitative literature review predominantly evaluated independent factors in exercising, encapsulated, and/or hot water immersed males. The most studied health conditions exacerbated by heat exposure were T2D and heart failure. Experimental designs that are prioritizing the very old, females in general, sex-specific differences, sustainable cooling strategies for older adults, and other known heat-sensitive diseases have been largely overlooked to date. Given forecasted global demographic changes, increasing prevalence of multimorbidity in older adults, and elevated frequency, intensity, and duration of heatwaves, future controlled experimental trials are needed to better our understanding of how various intersecting factors the physiological systems tasked with maintaining homeostasis during hot weather and heatwaves of varying characteristics. These controlled studies are required to better inform the direction of ecologically valid fieldwork studies such as evaluating the effectiveness of promising control measures for reducing heat health risk in vulnerable populations.

## Supplementary Material

Supplemental Material

## Data Availability

All associated data and the R code generated for analysis is available upon request.
