# Supplementary material for: Experimental research in environmentally induced hyperthermic older persons: A systematic quantitative literature review mapping the available evidence
Source: Temperature (Austin). Author manuscript; Available in PMC 2024 Apr 2. (PMC7615797; doi:10.1080/23328940.2023.2242062)
Supplement: Supplementary [file EMS194266-supplement-Supplementary.docx]

**Supplement**

**Table S1** Database search strings

| **Scopus (advanced)** |
| --- |
| (INDEXTERMS({aged}) OR INDEXTERMS({geriatric})OR TITLE-ABS-KEY({elderly}) OR INDEXTERMS(geriatrics) OR INDEXTERMS(elderly) OR TITLE-ABS-KEY({geriatric}) OR TITLE-ABS-KEY("older adult*") OR TITLE-ABS-KEY("older person*") OR TITLE-ABS-KEY("older people*")) AND (INDEXTERMS({Heat Stress Disorders}) OR INDEXTERMS({Body Temperature}) OR INDEXTERMS({Body Temperature Regulation}) OR TITLE-ABS-KEY({Body Temperature Regulation}) OR TITLE-ABS-KEY({Heat Stress}) OR TITLE-ABS-KEY({Body Temperature}) OR TITLE-ABS-KEY({Tympanic Temperature}) OR TITLE-ABS-KEY({Sublingual Temperature}) OR TITLE-ABS-KEY({Rectal Temperature}) OR TITLE-ABS-KEY("thermoregulat*") OR TITLE-ABS-KEY({thermal comfort}) OR INDEXTERMS({thermal comfort})) AND (TITLE-ABS-KEY({heatwave}) OR INDEXTERMS({heatwave}) OR INDEXTERMS({extreme heat}) OR TITLE-ABS-KEY({extreme heat}) OR TITLE-ABS-KEY({Hot Temperature}) OR INDEXTERMS({Hot Temperature})) |
| **PubMed** |
| (aged[Mesh] OR elderly[all] OR (geriatric[all] OR geriatrics[Mesh] OR geriatrics[all]) OR "older adult*"[all]) AND ("Heat Stress Disorders"[Mesh] OR "Body Temperature"[Mesh] OR ("Body Temperature Regulation"[Mesh] OR (body[all] AND temperature[all] AND regulation[all]) OR "Body Temperature Regulation"[all] OR thermoregulation[all] OR thermoregulate[all])) AND (heatwave[all] OR heatwaves[all] OR ("extreme heat"[Mesh] OR (extreme[all] AND heat[all]) OR "extreme heat"[all]) OR "Hot Temperature"[Mesh]) |
| **Web of Science** |
| (ALL=aged OR ALL=elderly OR ALL=geriatric OR ALL=geriatrics OR ALL="older adult*" OR ALL="older person*" OR ALL="older people*") AND (ALL="Heat Stress Disorders" OR ALL="Heat Stress" OR ALL="Body Temperature" OR ALL="Tympanic Temperature" OR ALL="Sublingual Temperature" OR ALL="Rectal Temperature" OR ALL="Body Temperature Regulation" OR ALL=thermoregulat*) AND (ALL=heatwave OR ALL=heatwaves OR ALL="extreme heat" OR ALL="Hot Temperature") |
| **CINAHL** |
| TX (MH aged+ OR TX "elderly" OR TX “geriatric” OR MH geriatrics+ OR TX “geriatrics” OR TX "older adult*") AND (MH "Heat Stress Disorders"+ OR MH "Body Temperature Regulation"+ OR MH "Body Temperature"+ OR TX (body AND temperature AND regulation) OR TX "Body Temperature Regulation" OR TX “thermoregulation” OR TX “thermoregulate”) AND (TX "heatwave" OR TX "heatwaves" OR MH "extreme heat"+ OR TX "extreme heat" OR MH "Hot Temperature"+) |
| **SportDiscus** |
| (DE "OLDER people" OR DE "OLDER people physiology" OR TX "elderly" OR TX “geriatric” OR TX “geriatrics” OR TX "older adult*") AND (DE "PHYSIOLOGICAL effects of heat" OR DE "HEAT adaptation" OR DE "HEAT exhaustion" OR DE "HEAT stroke" OR DE "BODY temperature regulation" OR DE "BODY temperature" OR DE "SKIN temperature" OR DE "TYMPANIC body temperature" OR TX (body AND temperature AND regulation) OR TX "Body Temperature Regulation" OR TX “thermoregulation” OR TX “thermoregulate”) AND (DE “HEAT” OR TX "heatwave" OR TX "heatwaves" OR TX "extreme heat") |

**Bibliography of all included studies**

Akerman, A. P. et al. Impact of uncomplicated controlled hypertension on thermoregulation during exercise-heat stress. *Journal of Human Hypertension* **35**, 880–883 (2021).

Amano, T., Fujii, N., Louie J. C., Meade, R. D. & Kenny, G. P. Individual variations in nitric oxide synthase-dependent sweating in young and older males during exercise in the heat: role of aerobic power. *Physiological Reports* **5**, e13208 (2017).

Amano, T. et al. Sweating responses to isometric hand-grip exercise and forearm muscle metaboreflex in prepubertal children and elderly. *Experimental Physiology* **102**, 214–227 (2017).

Andersen, I., Jensen, P. L., Junker, P., Thomsen A. & Wyon, D. P. The effects of moderate heat stress on patients with ischemic heart disease. *Scandinavian Journal of Work, Environment & Health* **2**, 256–268 (1976).

Anderson, R. K. & Kenney, W. L. Effect of age on heat-activated sweat gland density and flow during exercise in dry heat. *Journal of Applied Physiology* **63**, 1089–1094 (1987).

Armstrong, C. G. & Kenney, W. L. Effects of age and acclimation on responses to passive heat exposure. *Journal of Applied Physiology* **75**, 2162–2167 (1993).

Baker, L. B., Munce, T. A. & Kenney, W. L. Sex differences in voluntary fluid intake by older adults during exercise. *Medicine & Science in Sports & Exercise* **37**, 789–796 (2005).

Balmain, B.N. et al. Folic acid supplementation improves vascular endothelial function, yet not skin blood flow during exercise in the heat, in patients with heart failure. *American Journal of Physiology - Regulatory, Integrative and Comparative Physiology* **315**, R810–R819 (2018).

Balmain, B. N. et al. Altered thermoregulatory responses in heart failure patients exercising in the heat. *Physiological Reports* **4**, e13022 (2016).

Balmain, B. N. et al. Thermoeffector responses at a fixed rate of heat production in heart failure patients. *Medicine & Science in Sports & Exercise* **50**, 417–426 (2018).

Best, S., Caillaud, C. & Thompson, M. The effect of ageing and fitness on thermoregulatory response to high-intensity exercise. *Scandinavian Journal of Medicine & Science in Sports* **22**, e29-e37 (2012).

Best, S. et al. Exercise-heat acclimation in young and older trained cyclists. *Journal of Science and Medicine in Sport* **17**, 677–682 (2014).

Brazaitis, M. et al. Heat transfer and loss by whole-body hyperthermia during severe lower-body heating are impaired in healthy older men. *Experimental Gerontology* **96**, 12–18 (2017).

Brooks, E. M. et al. Chronic hormone replacement therapy alters thermoregulatory and vasomotor function in postmenopausal women. *Journal of Applied Physiology* **83**, 477–484 (1997).

Brooks-Asplund, E. M., Cannon, J. G. & Kenney, W. L. Influence of hormone replacement therapy and aspirin on temperature regulation in postmenopausal women. *American Journal of Physiology - Regulatory, Integrative and Comparative Physiology* **279**, R839-R848 (2000).

Bruning, R. S., Dahmus, J. D., Kenney, W. L. & Alexander, L. M. Aspirin and clopidogrel alter core temperature and skin blood flow during heat stress. *Medicine & Science in Sports & Exercise* **45**, 674–682 (2013).

Carrillo, A. E. et al. Heart rate variability during high heat stress: a comparison between young and older adults with and without Type 2 diabetes. *American Journal of Physiology - Regulatory, Integrative and Comparative Physiology* **311**, R669–R675 (2016).

Chia, E., Cannon, J. & Marino, F. E. The effects of acute versus chronic training status on pacing strategies of older men in a hot, humid environment. *Journal of Thermal Biology* **53**, 125–134 (2015).

Coull, N. A., West, A. M., Hodder, S. G., Wheeler, P. & Havenith, G. Body mapping of regional sweat distribution in young and older males. *European* *Journal of Applied Physiology* **121**, 109–125 (2021).

Cui, J. et al. Effects of heat stress on thermoregulatory responses in congestive heart failure patients. *Circulation* **112**, 2286–2292 (2005).

Cui, J. et al. Chronic heart failure does not attenuate the total activity of sympathetic outflow to skin during whole body heating. *Circulation: Heart Failure* **6**, 271–278 (2013).

Cui, J., Boehmer, J. P., Blaha, C. & Sinoway, L. I. Muscle sympathetic nerve activity response to heat stress is attenuated in chronic heart failure patients. *American Journal of Physiology - Regulatory, Integrative and Comparative Physiology* **312**, R873–R882 (2017).

Daanen, H. A. & Herweijer, J. A. Effectiveness of an indoor preparation program to increase thermal resilience in elderly for heat waves. *Building and Environment* **83**, 115–119 (2015).

de Korte, J. Q., Eijsvogels, T. M. H. , Hopman, M. T. E. , Bongers, C. C. W. G. Thermoregulatory, cardiovascular and perceptual responses of spectators of a simulated football match in hot and humid environmental conditions. *Sports* **11**, 78 (2023).

de Paula Viveiros, J., Amorim, F. T., Alves, M. N., Passos, R. L. & Meyer, F. Run performance of middle-aged and young adult runners in the heat. *International Journal of Sports Medicine* **33**, 211–217 (2012).

Dill, D. B. & Consolazio, C. F. Responses to exercise as related to age and environmental temperature. *Journal of Applied Physiology* **17**, 645–648 (1962).

Drinkwater, B. L., Bedi, J. F., Loucks, A. B., Roche, S. & Horvath, S. M. Sweating sensitivity and capacity of women in relation to age. *Journal of Applied Physiology: Respiratory, Environmental and Exercise Physiology* **53**, 671–676 (1982).

Dufour, A. & Candas, V. Ageing and thermal responses during passive heat exposure: sweating and sensory aspects. *European* *Journal of Applied Physiology* **100**, 19–26 (2007).

Dunbar, S. L. & Kenney, W. L. Effects of hormone replacement therapy on hemodynamic responses of postmenopausal women to passive heating. *Journal of Applied Physiology* **89**, 97–103 (2000).

Fennell, W. H. & Moore, R. E. Peripheral vascular responses to heat stress in elderly men. *International Journal of Biometeorology* **15**, 325–329 (1971).

Fennell, W. H. & Moore, R. E. Responses of aged men to passive heating. *The Journal of Physiology* **231**, 118P-119P (1973).

Fujii, N. et al. No effect of ascorbate on cutaneous vasodilation and sweating in older men and those with Type 2 diabetes exercising in the heat. *Physiological Reports* **5**, e13238 (2017).

Fujii, N. et al. iNOS-dependent sweating and eNOS-dependent cutaneous vasodilation are evident in younger adults, but are diminished in older adults exercising in the heat. *Journal of Applied Physiology* **120**, 318–327 (2016).

Fujii, N. et al. Do nitric oxide synthase and cyclooxygenase contribute to the heat loss responses in older males exercising in the heat? *The Journal of Physiology* **593**, 3169–3180 (2015).

Gagnon, D. et al. Age modulates physiological responses during fan use under extreme heat and humidity. *Medicine & Science in Sports & Exercise* **49**, 2333–2342 (2017).

Gagnon, D. et al. Folic acid supplementation does not attenuate thermoregulatory or cardiovascular strain of older adults exposed to extreme heat and humidity. *Experimental Physiology* **103**, 1123–1131 (2018).

Gagnon, D. et al. Volume loading augments cutaneous vasodilatation and cardiac output of heat stressed older adults. *The Journal of Physiology* **595**, 6489–6498 (2017).

Gagnon, D., Schlader, Z. J. & Crandall, C. G. Sympathetic activity during passive heat stress in healthy aged humans. *The Journal of Physiology* **593**, 2225–2235 (2015).

Gagnon, D. et al. Healthy aging does not compromise the augmentation of cardiac function during heat stress. *Journal of Applied Physiology* **121**, 885–892 (2016).

Gerrett, N., Amano, T., Inoue, Y. & Kondo, N. Eccrine sweat glands’ maximum ion reabsorption rates during passive heating in older adults (50-84 years). *European* *Journal of Applied Physiology* **121**, 3145–3159 (2021).

Grassi, G. et al. Impairment of thermoregulatory control of skin sympathetic nerve traffic in the elderly. Circulation **108**, 729–735 (2003).

Greaney, J. L., Stanhewicz, A. E., Proctor, D. N., Alexander, L. M. & Kenney, W. L. Impairments in central cardiovascular function contribute to attenuated reflex vasodilation in aged skin. *Journal of Applied Physiology* **119**, 1411–1420 (2015).

Green, D. J. et al. Impaired skin blood flow response to environmental heating in chronic heart failure. *European Heart Journal* **27**, 338–343 (2006).

Henschel, A., Cole, M., Lyczkowskyi, O., Doyle, T. & Margolies, L. Heat tolerance of elderly persons living in a subtropical climate. *Journal of Gerontology* **23**, 17-22 (1967).

Ho, C. W., Beard, J. L., Farrell, P. A., Minson, C. T. & Kenney, W. L. Age, fitness, and regional blood flow during exercise in the heat. *Journal of Applied Physiology* **82**, 1126–1135 (1997).

Holowatz, L. A., Jennings, J. D., Lang, J. A. & Kenney, W. L. Ketorolac alters blood flow during normothermia but not during hyperthermia in middle-aged human skin. *Journal of Applied Physiology* **107**, 1121–1127 (2009).

Holowatz, L. A., Thompson, C. S. & Kenney, W. L. Acute ascorbate supplementation alone or combined with arginase inhibition augments reflex cutaneous vasodilation in aged human skin. *American Journal of Physiology - Heart and Circulatory Physiology* **291**, 2965–2970 (2006).

Holowatz, L. A., Jennings, J. D., Lang, J. A. & Kenney, W. L. Systemic low-dose aspirin and clopidogrel independently attenuate reflex cutaneous vasodilation in middle-aged humans. *Journal of Applied Physiology* **108**, 1575–1581 (2010).

Inbar, O., Morris, N., Epstein, Y. & Gass, G. Comparison of thermoregulatory responses to exercise in dry heat among prepubertal boys, young adults and older males. *Experimental Physiology* **89**, 691–700 (2004).

Inoue, Y., Havenith, G., Kenney, W. L., Loomis, J. L. & Buskirk, E. R. Exercise- and methylcholine-induced sweating responses in older and younger men: effect of heat acclimation and aerobic fitness. *International Journal of Biometeorology* **42**, 210–216 (1999).

Inoue Y., Nakao, M., Araki, T. & Murakami, H. Regional differences in the sweating responses of older and younger men. *Journal of Applied Physiology* **71**, 2453–2459 (1991).

Inoue, Y. & Shibasaki, M. Regional differences in age-related decrements of the cutaneous vascular and sweating responses to passive heating. *European Journal of Applied Physiology and Occupational Physiology* **74**, 78–84 (1996).

Inoue, Y., Shibasaki, M., Ueda, H. & Ishizashi. H. Mechanisms underlying the age-related decrement in the human sweating response. *European Journal of Applied Physiology and Occupational Physiology* **79**, 121–126 (1999).

Inoue, Y., Nakao, M., Okudaira, S., Ueda, H. & Araki, T. Seasonal variation in sweating responses of older and younger men. *European Journal of Applied Physiology and Occupational Physiology* **70**, 6–12 (1995).

Inoue, Y., Shibasaki, M., Hirata, K. & Araki, T. Relationship between skin blood flow and sweating rate, and age related regional differences. *European Journal of Applied Physiology and Occupational Physiology* **79**, 17–23 (1998).

James, T. J., et al. Timing of acute passive heating on glucose tolerance and blood pressure in people with type 2 diabetes: a randomized, balanced crossover, control trial. *Journal of Applied Physiology* **130**, 1093-1105 (2021).

Journeay, W. S., et al. Impacts of age, diabetes, and hypertension on serum endothelial monocyte‐activating polypeptide‐II after prolonged work in the heat. *American Journal of Industrial Medicine* ***66***, 610-619 (2023).Kemp, J., Després, O., Pebayle, T. & Dufour, A. Age-related decline in thermal adaptation capacities: an evoked potentials study. *Psychophysiology* **51**, 539–545 (2014).

Kenney, W. L. & Anderson, R. K. Responses of older and younger women to exercise in dry and humid heat without fluid replacement. *Medicine & Science in Sports & Exercise* **20**, 155–160 (1988).

Kenney, W. L. Control of heat-induced cutaneous vasodilatation in relation to age. *European Journal of Applied Physiology and Occupational Physiology* **57**, 120–125 (1988).

Kenney, W. L. Psychrometric limits and critical evaporative coefficients for exercising older women. *Journal of Applied Physiology* **129**, 263–271 (2020).

Kenney, W. L. et al. Age and hypohydration independently influence the peripheral vascular response to heat stress. *Journal of Applied Physiology* **68**, 1902–1908 (1990).

Kenney, W. L. & Ho, C. Age alters regional distribution of blood flow during moderate-intensity exercise. *Journal of Applied Physiology* **79**, 1112–1119 (1995).

Kenney, W. L. et al. Decreased active vasodilator sensitivity in aged skin. *American Journal of Physiology - Heart and Circulatory Physiology* **272**, H1609-H1614 (1997).

Kenney, W. L. & Zappe, DH. Effect of age on renal blood flow during exercise. *Aging* **6**, 293–302 (1994).

Kenny, G. P. et al. Hyperthermia and cardiovascular strain during an extreme heat exposure in young versus older adults. *Temperature* **4**, 79–88 (2017).

Kenny, G. P., Stapleton, J. M., Yardley, J. E., Boulay, P. & Sigal, R. J. Older adults with Type 2 diabetes store more heat during exercise. *Medicine & Science in Sports & Exercise* **45**, 1906–1914 (2013).

Kenny, G. P. et al. Older adults experience greater levels of thermal and cardiovascular strain during extreme heat exposures. *Medicine & Science in Sports & Exercise* **47**, 497 (2015).

Kenny, G. P. et al. Older firefighters are susceptible to age-related impairments in heat dissipation. *Medicine & Science in Sports & Exercise* **47**, 1281–1290 (2015).

Kihara, M., Sugenoya, J. & Takahashi, A. The assessment of sudomotor dysfunction in multiple system atrophy. *Clinical Autonomic Research* 1, 297–302 (1991).

King, K. E., McCormick, J. J., Notley, S. R., Fujii, N. & Kenny, G. P. Serum klotho concentrations in young and older men during prolonged exercise in temperate and hot conditions. *Current Aging Science* **15**, 180–185 (2022).

King, K. E., et al. Serum klotho concentrations in older men with hypertension or type 2 diabetes during prolonged exercise in temperate and hot conditions. *European Journal of Applied Physiology* ***123****,*1519-1527 (2023).

Larose, J. et al. Age-related differences in heat loss capacity occur under both dry and humid heat stress conditions. *Journal of Applied Physiology* **117**, 69–79 (2014).

Larose, J. et al. Do older females store more heat than younger females during exercise in the heat? *Medicine & Science in Sports & Exercise* **45**, 2265–2276 (2013).

Larose, J., Boulay, P., Sigal, RJ., Wright, H. E. & Kenny, G. P. Age-related decrements in heat dissipation during physical activity occur as early as the age of 40. *PloS one* **8**, e83148 (2013).

Larose, J. et al. Whole body heat loss is reduced in older males during short bouts of intermittent exercise. *American Journal of Physiology - Regulatory, Integrative and Comparative Physiology* **305**, R619-R629 (2013).

Lucas, R. A. I., Cotter, J. D., Morrison, S. & Ainslie, P. N. The effects of ageing and passive heating on cardiorespiratory and cerebrovascular responses to orthostatic stress in humans. *Experimental Physiology* **93**, 1104–1117 (2008).

Lucas, R. A., Ainslie, P. N., Morrison, S. A. & Cotter, J. D. Compression leggings modestly affect cardiovascular but not cerebrovascular responses to heat and orthostatic stress in young and older adults. *Age* **34**, 439–449 (2012).

Lucas, R. A. I., Sarma, S., Schlader, Z. J., Pearson, J. & Crandall, C. G. Age‐related changes to cardiac systolic and diastolic function during whole‐body passive hyperthermia. *Experimental Physiology* **100**, 422–434 (2015).

Macartney, M. J. et al. Diminished heart rate variability in Type 2 diabetes is exacerbated during exercise-heat stress. *Acta Diabetologica* **57**, 899–901 (2020).

Macartney, M. J. et al. Effect of exercise-heat acclimation on cardiac autonomic modulation in Type 2 diabetes: a pilot study. *Applied Physiology, Nutrition & Metabolism* **46**, 284–287 (2021).

Mack, G. W. et al. Body fluid balance in dehydrated healthy older men: thirst and renal osmoregulation. *Journal of Applied Physiology* **76**, 1615–1623 (1994).

Marszalek, A. Thirst and work capacity of older people in a hot environment. *International Journal of Occupational Safety and Ergonomics* **6**, 135–142 (2000).

McCormick, J. J. et al. Exercise in the heat induces similar elevations in serum irisin in young and older men despite lower resting irisin concentrations in older adults. *Journal of Thermal Biology* **104**, 103189 (2022).

McCormick, J. J. et al. The serum irisin response to prolonged physical activity in temperate and hot environments in older men with hypertension or type 2 diabetes. *Journal of Thermal Biology* ***110*** 103344 (2022).

McGarr, G. W. et al. Superoxide and NADPH oxidase do not modulate skin blood flow in older exercising adults with and without Type 2 diabetes. *Microvascular Research* **125**, 103886 (2019).

McLellan, K., et al. Multiple stressors and the response of vascular endothelial cells: the effect of aging and diabetes. *Diabetes Technology & Therapeutics* **11**, 73–79 (2009).

Meade, R. D., Notley, S. R., Rutherford, M. M., Boulay, P. & Kenny, G. P. Ageing attenuates the effect of extracellular hyperosmolality on whole-body heat exchange during exercise-heat stress. *The Journal of Physiology* **598**, 5133–5148 (2020).

Meade, R. D. et al. Efficacy of cooling centers for mitigating physiological strain in older adults during daylong heat exposure: A laboratory-based heat wave simulation. *Environmental Health Perspectives* **131**, 067003 (2023).

Meade, R. D. et al. Interactive effects of age and hydration state on human thermoregulatory function during exercise in hot‐dry conditions. *Acta Physiologica* **226**, e13226 (2019).

Miescher, E. & Fortney, S. M. Responses to dehydration and rehydration during heat exposure in young and older men. *American Journal of Physiology - Regulatory, Integrative and Comparative Physiology* **257**, R1050-R1056 (1989).

Minson, C. T, Wladkowski S. L., Cardell, A. F., Pawelczyk, J. A. & Kenney, W. L. Age alters the cardiovascular response to direct passive heating. *Journal of Applied Physiology* **84**, 1323–1332 (1998).

Minson, C. T., Wladkowski, S. L., Pawelczyk, J. A. & Kenney, W. L. Age, splanchnic vasoconstriction, and heat stress during tilting. *American Journal of Physiology - Regulatory, Integrative and Comparative Physiology* **276**, R203-R212 (1999).

Minson, C. T. & Kenney, W. L. Age and cardiac output during cycle exercise in thermoneutral and warm environments. *Medicine & Science in Sports & Exercise* **29**, 75–81 (1997).

Nagasawa, Y. et al. Effects of hot bath immersion on autonomic activity and hemodynamics: comparison of the elderly patient and the healthy young. *Japanese Circulation Journal* **65**, 587–592 (2001).

Natsume, K., Ogawa, T., Sugenoya, J., Ohnishi, N. & Imai, K. Preferred ambient temperature for old and young men in summer and winter. *International Journal of Biometeorology* **36**, 1–4 (1992).

Notley, S. R. et al. Heat tolerance and occupational heat exposure limits in older men with and without Type 2 diabetes or hypertension. *Medicine & Science in Sports & Exercise* **53**, 2196-2206 (2021).

Notley, S. R. et al. Evidence for age-related differences in heat acclimatisation responsiveness. *Experimental Physiology* **105**, 1491–1499 (2020).

Notley, S. R. et al. Heat exchange in young and older men during constant- and variable-intensity work. *Medicine & Science in Sports & Exercise* **52**, 2628–2636 (2020).

Notley, S. R. et al. Aging impairs whole-body heat loss in women under both dry and humid heat stress. *Medicine & Science in Sports & Exercise* **49**, 2324–2332 (2017).

Notley, S. R. et al. Exercise-heat tolerance in middle-aged-to-older men with Type 2 diabetes. *Acta Diabetologica* **58**, 809–812 (2021).

Notley, S. R. et al. Exercise heat stress in patients with and without Type 2 diabetes. *JAMA* **322**, 1409–1411 (2019).

Okazaki, K. et al. Effects of exercise training on thermoregulatory responses and blood volume in older men. *Journal of Applied Physiology* **93**, 1630–1637 (2002).

Petrofsky, J. S., Besonis, C., Rivera, D., Schwab, E. & Lee, S. Impairment in orthostatic tolerance during heat exposure in individuals with Type I and Type II diabetes. *Medical Science Monitor* **11**, CR153-CR159 (2005).

Petrofsky, J. S., Lee, S. & Cuneo-Libarona, M. The impact of rosiglitazone on heat tolerance in patients with Type 2 diabetes. *Medical Science Monitor* **11**, CR562-CR569 (2005).

Petrofsky, J. S. et al. The effect of aging on conductive heat exchange in the skin at two environmental temperatures. *Medical Science Monitor* **12**, CR400-CR408 (2006).

Petrofsky, J. S., Besonis, C. & Rivera, D. Circulatory response to hydrotherapy and dry heat in individuals with Type 2 diabetes. *International Journal of Therapy and Rehabilitation* **12**, 491–497 (2005).

Petrofsky, J. S., Lee, S. W., Cuneo-Libarona, M. & Apodaca, P. The effect of rosiglitazone on orthostatic tolerance during heat exposure in individuals with Type II diabetes. *Diabetes Technology & Therapeutics* **9**, 377–386 (2007).

Pierzga, J. M., Frymoyer, A. & Kenney, W. L. Delayed distribution of active vasodilation and altered vascular conductance in aged skin. *Journal of Applied Physiology* **94**, 1045–1053 (2002).

Poirier, M. P. et al. Type 2 diabetes does not exacerbate body heat storage in older adults during brief, extreme passive heat exposure. *Temperature* **7**, 263–269 (2020).

Relf, R. L. et al. Thermoregulation is not impaired in breast cancer survivors during moderate-intensity exercise performed in warm and hot environments. *Physiological Reports* **9**, e14968 (2021).

Robinson, S., Belding, H. S., Consolazio, F. C., Horvath, S. M. & Turrell, E. S. Acclimatization of older men to work in heat. *Journal of Applied Physiology* **20**, 583–586 (1965).

Romero, S. A. et al. Acute limb heating improves macro- and microvascular dilator function in the leg of aged humans. *American Journal of Physiology - Heart and Circulatory Physiology* **312**, H89–H97 (2016).

Sagawa, S., Shiraki, K., Yousef, M. K. & Miki, K. Sweating and cardiovascular responses of aged men to heat exposure. *Journal of Gerontology* **43**, M1-M8 (1988).

Schlader, Z. J. et al. Cognitive and perceptual responses during passive heat stress in younger and older adults. *American Journal of Physiology - Regulatory, Integrative and Comparative Physiology* **308**, R847-

Schmidt, M. D. et al. Revisiting regional variation in the age-related reduction in sweat rate during passive heat stress. *Physiological Reports* **10**, e15250 (2022).

Scremin, G. & Kenney, W. L. Aging and the skin blood flow response to the unloading of baroreceptors during heat and cold stress. *Journal of Applied Physiology* **96**, 1019–1025 (2004).

Shiraki, K., Sagawa, S., Yousef, M. K., Konda, N. & Miki, K. Physiological responses of aged men to head-up tilt during heat exposure. *Journal of Applied Physiology* **63**, 576–581 (1987).

Shoenfeld, Y., Udassin, R., Shapiro, Y., Ohri, A. & Sohar, E. Age and sex difference in response to short exposure to extreme dry heat. *Journal of Applied Physiology: Respiratory, Environmental and Exercise Physiology* **44**, 1–4 (1978).

Smith, C.J., Alexander, L.M. & Kenney, W. L. Nonuniform, age-related decrements in regional sweating and skin blood flow. *American Journal of Physiology - Regulatory, Integrative and Comparative Physiology* **305**, R877-R885 (2013).

Smolander, J., Korhonen, O. & Ilmarinen, R. Responses of young and older men during prolonged exercise in dry and humid heat. *European Journal of Applied Physiology and Occupational Physiology* **61**, 413–418 (1990).

Sokolnicki, L. A. et al. Skin blood flow and nitric oxide during body heating in Type 2 diabetes mellitus. *Journal of Applied Physiology* **106**, 566–570 (2009).

Stanhewicz, A. E., Alexander, L. M. & Kenney, W. L. Oral sapropterin acutely augments reflex vasodilation in aged human skin through nitric oxide-dependent mechanisms. *Journal of Applied Physiology* **115**, 972–978 (2013).

Stanhewicz, A. E., Bruning, R. S., Smith, C. J., Kenney, W. L. & Holowatz, L. A. Local tetrahydrobiopterin administration augments reflex cutaneous vasodilation through nitric oxide-dependent mechanisms in aged human skin. *Journal of Applied Physiology* **112**, 791–797 (2011).

Stanhewicz, A. E., Greaney, J. L., Alexander, L. M. & Kenney, W. L. Folic acid supplementation increases cutaneous vasodilator sensitivity to sympathetic nerve activity in older adults. *American Journal of Physiology - Regulatory, Integrative and Comparative Physiology* 312, R681–R688 (2017).

Stapleton, J. M. et al. Do older adults experience greater thermal strain during heat waves? *Applied Physiology, Nutrition & Metabolism* **39**, 292–298 (2014).

Stapleton, J. M., Fujii, N., Carter, M. & Kenny, G. P. Diminished nitric oxide-dependent sweating in older males during intermittent exercise in the heat. *Experimental Physiology* **99**, 921–932 (2014).

Stapleton, J. M. et al. Aging impairs heat loss, but when does it matter? *Journal of Applied Physiology* **118**, 299–309 (2015).

Stapleton, J. M. et al. At what level of heat load are age-related impairments in the ability to dissipate heat evident in females? *PLoS One* **10**, e0119079 (2015).

Stapleton, J. M., Fujii, N., McGinn, R., McDonald, K. & Kenny, GP. Age-related differences in postsynaptic increases in sweating and skin blood flow postexercise. *Physiological Reports* **2**, e12078 (2014).

Steward, C. J., et al. The effect of age and mitigation strategies during hot water immersion on orthostatic intolerance and thermal stress. *Experimental Physiology* **108**, 554-567 (2023).

Takamata, A. et al. Effect of an exercise-heat acclimation program on body fluid regulatory responses to dehydration in older men. *American Journal of Physiology - Regulatory, Integrative and Comparative Physiology* **277**, R1041–R1050 (1999).

Takeda, R. et al. Lower thermal sensation in normothermic and mildly hyperthermic older adults. *European* *Journal of Applied Physiology* **116**, 975–984 (2016).

Tam, H., Frewin, D., Elliott, K., Luke, W. & Downey, J. The effect of aspirin pretreatment on the sweating response of older female subjects. *The Australian Journal of Experimental Biology and Medical Science* **55**, 225–228 (1977).

Tankersley, C. G., Smolander, J., Kenney, W. L. & Fortney, S. M. Sweating and skin blood flow during exercise: effects of age and maximal oxygen uptake. *Journal of Applied Physiology* **71**, 236–242 (1991).

Thomas, C. M., Pierzga, J. M. & Kenney, W. L. Aerobic training and cutaneous vasodilation in young and older men. *Journal of Applied Physiology* **86**, 1676–1686 (1999).

Tokizawa, K. Effects of wetted inner clothing on thermal strain in young and older males while wearing ventilation garments. *Frontiers in Physiology* **14**, 1122504 (2023).

Ueno, S., Ikeda, K. & Tai, T. Metabolic rate prediction in young and old men by heart rate, ambient temperature, weight and body fat percentage. *Journal of Occupational Health* **56**, 519–525 (2014).

Wagner, J. A., Robinson, S., Tzankoff, S. P. & Marino, R. P. Heat tolerance and acclimatization to work in the heat in relation to age. *Journal of Applied Physiology* **33**, 616–622 (1972).

Waldock, K. A. M. et al. Exercise heat acclimation and post-exercise hot water immersion improve resting and exercise responses to heat stress in the elderly. *Journal of Science & Medicine in Sport* **24**, 774–780 (2021).

Wick, D. E. et al. Delayed threshold for active cutaneous vasodilation in patients with Type 2 diabetes mellitus. *Journal of Applied Physiology* **100**, 637–641 (2006).

Wright, H. E. et al. Moderate-intensity intermittent work in the heat results in similar low-level dehydration in young and older males. *Journal of Occupational and Environmental Hygiene* **11**, 144–153 (2014).

Wright, H. E. et al. Are circulating cytokine responses to exercise in the heat augmented in older men? *Applied Physiology, Nutrition & Metabolism* **39**, 117–123 (2014).

Wright-Beatty, H. E., Hardcastle, S. G., Boulay, P., Flouris, A. D. & Kenny, G. P. Increased air velocity reduces thermal and cardiovascular strain in young and older males during humid exertional heat stress. *Journal of Occupational and Environmental Hygiene* **12**, 625–634 (2015).

Wright-Beatty, H. E., Hardcastle, S. G., Boulay, Larose, J. & Kenny, G. P. Increased air velocity during exercise in the heat leads to equal reductions in hydration shifts and interleukin-6 with age. *European* *Journal of Applied Physiology* **114**, 2081–2092 (2014).

Wright-Beatty, H. E. et al. Inflammatory responses of older firefighters to intermittent exercise in the heat. *European* *Journal of Applied Physiology* **114**, 1163–1174 (2014).

Zappe, D. H., Bell, G. W., Swartzentruber, H., Wideman, R. F. & Kenney, W. L. Age and regulation of fluid and electrolyte balance during repeated exercise sessions. *American Journal of Physiology - Regulatory, Integrative and Comparative Physiology* **270**, R71-R79 (1996).

**List of all excluded studies (full-text screening)**

Abdelmoety DA; El-Bakri NK; Almowalld WO; Turkistani ZA; Bugis BH; Baseif EA; Melbari MH; AlHarbi K; Abu-Shaheen A. 2018. Characteristics Of Heat Illness During Hajj: A Cross-Sectional Study. Biomed Res Int. DOI:10.1155/2018/5629474

Allison TG; Miller TD; Squires RW; Gau GT. 1993. Cardiovascular Responses To Immersion In A Hot Tub In Comparison With Exercise In Male Subjects With Coronary Artery Disease. Mayo Clin Proc. DOI:10.1016/s0025-6196(12)60014-7

Alt E; Hirgstetter C; Heinz M; Theres H; Blomer H. 1988. Central Venous Blood Temperature For Rate Control Of Physiological Pacemakers. J Cardiovasc Surg.

Ambrosi P; Villani P; Bouvenot G. 2004. Hyponatremia In Elderly Patients Treated With Thiazide Diuretics And Incited To Drink Abundantly During The Heat Wave. Presse Med. DOI:10.1016/s0755-4982(04)98657-5

Anderson G S; Meneilly GS; Mekjavic IB. 1996. Passive Temperature Lability In The Elderly. Eur J Appl Physiol.

Andrews GR; Ofner F. 1972. The Limitations Of Reflex Heating Therapy In The Elderly. J Am Geriatr Soc. DOI:10.1111/j.1532-5415.1972.tb00774.x

Ansari A; Burch GE. 1969. Influence Of Hot Environments On The Cardiovascular System A Clinical Study Of 23 Cardiac Patients At Rest. Arch Intern Med.

Araki T; Toda Y; Matsushita K; Tsujino A. 1979. Age Differences In Sweating During Muscular Exercise. Jpn J Phys Fit Sports Med. DOI:10.7600/jspfsm1949.28.239

Arbab-Zadeh A; Crandall CG; Levine BD. 2002. Thermoregulation In Patients With Cardiac Disease. J Cardiopulm Rehabil. DOI:10.1097/00008483-200201000-00005

Arcury TA; Summers P; Talton JW; Chen H; Sandberg JC; Spears Johnson CR; Quandt SA. 2015. Heat Illness Among North Carolina Latino Farmworkers. J Occup Environ Med. DOI:10.1097/JOM.0000000000000552

Argaud L; Ferry T; Le QH; Marfisi A; Ciorba D; Achache P; Ducluzeau R; Robert D. 2007. Short- And Long-Term Outcomes Of Heatstroke Following The 2003 Heat Wave In Lyon, France. Arch Intern Med. DOI:10.1001/archinte.167.20.ioi70147

Ashraf A; Roshanzamir S; Bemana G; Mohammadi A; Jahani N; Naseri M. 2015. Sympathetic Skin Response And Vasomotor Symptoms In Postmenopausal Osteoporotic Women. Ijcbnm.

Auger N; Rheaume MA; Bilodeau-Bertrand M; Tang T; Kosatsky T. 2017. Climate And The Eye: Case-Crossover Analysis Of Retinal Detachment After Exposure To Ambient Heat. Environ Res. DOI:10.1016/j.envres.2017.05.017

Austin KG; Hansbrough JF; Dore C; Noordenbos J; Buono MJ. 2003. Thermoregulation In Burn Patients During Exercise. J Burn Care Rehabil. DOI:10.1097/00004630-200301000-00004

Avila S; Buono MJ. 2012. Priming Of The Sweat Glands Explains Reflex Sweating In The Heat. Int J Hyperthermia. DOI:10.3109/02656736.2011.613891

Badyshtov BA; Sytnik SI; Pastushenkov VA; Losev AS; Kolotilinskaia NV; Makhnycheva AL; Potapova LM; Seredenin SB. 1993. Various Reactions To Thermal Exposure In People With Varying Levels Of Thermal Resistance. Fiziol Cheloveka.

Baillot M; Le Bris S; Hue O. 2014. Fluid Replacement Strategy During A 27-Km Trail Run In Hot And Humid Conditions. Int J Sports Med. DOI:10.1055/s-0033-1349108

Barbieri A; Pinna C; Fruggeri L; Biagioni E; Campagna A. 2006. Heat Wave In Italy And Hyperthermia Syndrome. South Med J. DOI:10.1097/01.smj.0000231244.54790.d8

Bartlett G; Stewart JD; Tamblyn R; Abrahamowicz M. 1998. Normal Distributions Of Thermal And Vibration Sensory Thresholds. Muscle Nerve. DOI:10.1002/(sici)1097-4598(199803)21:3<367::aid-mus11>3.0.co;2-x

Bartnicki C; Ejsmont W; Dubrawski R. 1969. Differences Of Some Physiological Reactions In Women And Men Exposed To The Effect Of High Environmental Temperature. Biul Inst Med Morsk Gdansk.

Bartnicki C; Waskiewicz J; Dubrawski R. 1969. Behaviour Of Some Physiological Indexes Depending On The Age Of People Exposed To Short Action Of Humid Heat Under Experimental Conditions. Biul Inst Med Morsk Gdansk.

Basu R; Pearson D; Malig B; Broadwin R; Green R. 2012. The Effect Of High Ambient Temperature On Emergency Room Visits. Epidemiology. DOI:10.1097/EDE.0b013e31826b7f97

Basu R; Samet JM. 2002. An Exposure Assessment Study Of Ambient Heat Exposure In An Elderly Population In Baltimore, Maryland. Environ Health Perspect. DOI:10.1289/ehp.021101219

Belmin J; Auffray JC; Berbezier C; Boirin P; Mercier S; de Reviers B; Golmard JL. 2007. Level Of Dependency: A Simple Marker Associated With Mortality During The 2003 Heatwave Among French Dependent Elderly People Living In The Community Or In Institutions. Age Ageing. DOI:10.1093/ageing/afm026

Belval LN; Giersch GEW; Adams WM; Hosokawa Y; Jardine JF; Katch RK; Stearns RL; Casa DJ. 2020. Age- And Sex-Based Differences In Exertional Heat Stroke Incidence In A 7-Mile Road Race. J Athl Train. DOI:10.4085/1062-6050-539-19

Berenson GS; Burch GE. 1952. The Response Of Patients With Congestive Heart Failure To A Rapid Elevation In Atmospheric Temperature And Humidity. Am J Med Sci. DOI:10.1097/00000441-195201000-00008

Bernhardt JM; Breakey S; Cox R; Olayinka O; Quinn L; Simmonds K; Atkin K; Sipe M; Nicholas PK. 2023. Development Of A Screening Tool For Assessment Of Climate Change-Related Heat Illness In The Clinical Setting. J Am Assoc Nurse Pract. DOI:10.1097/JXX.0000000000000856

Bertelsmann FW; Heimans JJ; Weber EJ; van der Veen EA; Schouten JA. 1985. Thermal Discrimination Thresholds In Normal Subjects And In Patients With Diabetic Neuropathy. J Neurol Neurosurg Psychiatry. DOI:10.1136/jnnp.48.7.686

Blau JN; Engel HO. 1999. A New Cluster Headache Precipitant: Increased Body Heat. Lancet. DOI:10.1016/s0140-6736(99)01972-8

Bongers KS; Salahudeen MS; Peterson GM. 2020. Drugs Associated Hyperthermia: A Longitudinal Analysis Of Hospital Presentations. J Clin Pharm Ther. DOI:10.1111/jcpt.13090

Boonruksa P; Maturachon T; Kongtip P; Woskie S. 2020. Heat Stress, Physiological Response, And Heat-Related Symptoms Among Thai Sugarcane Workers. Int J Environ Res Public Health. DOI:10.3390/ijerph17176363

Bovy P; Foidart M; Dequinze B; Solheid M; Pirnay F. 1990. The Effect Of Hot Baths On The Muscular Properties Of Healthy And Of Spastic Subjects. Acta Belg Med Phys.

Brearley MB; Norton I; Rush D; Hutton M; Smith S; Ward L; Fuentes H. 2016. Influence Of Chronic Heat Acclimatization On Occupational Thermal Strain In Tropical Field Conditions. J Occup Environ Med. DOI:10.1097/JOM.0000000000000902

Brengelmann GL; Savage MV; Avery DH. 1994. Reproducibility Of Core Temperature Threshold For Sweating Onset In Humans. J Appl Physiol. DOI:10.1152/jappl.1994.77.4.1671

Buono MJ; McKenzie BK; Kasch FW. 1991. Effects Of Ageing And Physical Training On The Peripheral Sweat Production Of The Human Eccrine Sweat Gland. Age Ageing. DOI:10.1093/ageing/20.6.439

Burch GE. 1946. The Influence Of Environmental Temperature And Relative Humidity On The Rate Of Water Loss Through The Skin In Congestive Heart Failure In A Subtropical Climate. Am J Med Sci. DOI:10.1097/00000441-194602000-00008

Burch GE; DePasquale NP. 1968. Heat Humidity And Heart Disease. Postgrad Med J. DOI:10.1080/00325481.1968.11693393

Burch GE; DePasquale NP. 1962. Hot Climates, Man And His Heart. Book.

Burch GE; Hyman AL. 1957. Influence Of A Hot And Humid Environment Upon Cardiac Output And Work In Normal Man And In Patients With Chronic Congestive Heart Failure At Rest. Am Heart J. DOI:10.1016/0002-8703(57)90336-8

Burch GE; Miller GC. 1969. The Effects Of Warm, Humid Environment On Patients With Congestive Heart Failure. South Med J. DOI:10.1097/00007611-196907000-00012

Buskirk ER. 1977. Temperature Regulation With Exercise. Exerc Sport Sci Rev.

Caldwell AR; Robinson FB; Tucker MA; Arcement CH; Butts CL; McDermott BP; Ganio MS. 2017. Effect Of Passive Heat Stress And Exercise In The Heat On Arterial Stiffness. Eur J Appl Physiol. DOI:10.1007/s00421-017-3658-1

Calkins MM; Bonauto D; Hajat A; Lieblich M; Seixas N; Sheppard L; Spector JT. 2019. A Case-Crossover Study Of Heat Exposure And Injury Risk Among Outdoor Construction Workers In Washington State. Scand J Work Environ Health. DOI:10.5271/sjweh.3814

Charkoudian N. 2017. Getting Help From Frank And Starling (And Coats And Bowditch) To Augment Blood Flow In Heat-Stressed Older Adults. J Physiol. DOI:10.1113/JP275135

Chaseling GK; Filingeri D; Barnett M; Hoang P; Davis SL; Jay O. 2018. Cold Water Ingestion Improves Exercise Tolerance Of Heat-Sensitive People With Ms. Med Sci Sports Exerc. DOI:10.1249/MSS.0000000000001496

Chindapol S; Blair J; Osmond P; Prasad D. 2017. A Suitable Thermal Stress Index For The Elderly In Summer Tropical Climates. Ihbe 2016. DOI:10.1016/j.proeng.2017.04.253

Cornali C; Franzoni S; Riello R; Ghianda D; Frisoni GB; Trabucchi M. 2004. Effect Of High Climate Temperature On The Behavioral And Psychological Symptoms Of Dementia. J Am Med Dir Assoc. DOI:10.1097/01.JAM.0000126422.82173.87

Cramer MN; Huang M; Hieda M; Moralez G; Crandall CG. 2019. Effect Of Dietary Nitrate Supplementation With Beet Root Juice On Thermoregulatory And Cardiovascular Responses To Extreme Heat In Aged Humans. Med Sci Sports Exerc. DOI:10.1249/01.mss.0000562211.68536.fe

Crandall CG. 2000. Carotid Baroreflex Responsiveness In Heat-Stressed Humans. Am J Physiol Heart Circ Physiol .

Crowe J; Nilsson M; Kjellstrom T; Wesseling C. 2015. Heat-Related Symptoms In Sugarcane Harvesters. Am J Ind Med. DOI:10.1002/ajim.22450

Crowe JP; Moore RE. 1974. Proceedings: Physiological And Behavioural Responses Of Aged Men To Passive Heating. J Physiol.

Cui J; Wilson TE; Crandall CG. 2004. Orthostatic Challenge Does Not Alter Skin Sympathetic Nerve Activity In Heat-Stressed Humans. Auton Neurosci. DOI:10.1016/j.autneu.2004.08.009

Culp K; Tonelli S. 2019. Heat-Related Illness In Midwestern Hispanic Farmworkers: A Descriptive Analysis Of Hydration Status And Reported Symptoms. Workplace Health Saf. DOI:10.1177/2165079918813380

Cuthbertson DP; Rahimi AG. 1973. Metabolism After Injury 2 Effect Of A Dryish And Warm Environment On Skin Temperatures And Electrolyte Responses. Br J Surg. DOI:10.1002/bjs.1800600602

Dang BN; Dowell CH. 2014. Factors Associated With Heat Strain Among Workers At An Aluminum Smelter In Texas. J Occup Environ Med. DOI:10.1097/JOM.0000000000000095

Davido A; Patzak A; Dart T; Sadier MP; Meraud P; Masmoudi R; Sembach N; Cao TH. 2006. Risk Factors For Heat Related Death During The August 2003 Heat Wave In Paris, France, In Patients Evaluated At The Emergency Department Of The Hopital Europeen Georges Pompidou. Emerg Med J. DOI:10.1136/emj.2005.028290

Dill DB; Hall FG; Van Beaumont W. 1966. Sweat Chloride Concentration: Sweat Rate, Metabolic Rate, Skin Temperature, And Age. J Appl Physiol. DOI:10.1152/jappl.1966.21.1.99

Dill DB; Yousef MK; Nelson JD. 1973. Responses Of Men And Women To Two-Hour Walks In Desert Heat. J Appl Physiol. DOI:10.1152/jappl.1973.35.2.231

Dodman B; Cunliffe WJ; Roberts BE; Buchan CW. 1973. Effects Of Changes In Temperature (Local And Central) On Plasma Fibrinolytic Activity. J Clin Pathol. DOI:10.1136/jcp.26.4.248

Downey JA; LeRoy EC; Miller JM 3rd; Darling RC. 1971. Thermoregulation And Raynaud'S Phenomenon. Clin Sci. DOI:10.1042/cs0400211

Drummond PD; Finch PM. 1989. Reflex Control Of Facial Flushing During Body Heating In Man. Brain. DOI:10.1093/brain/112.5.1351

D'Souza AW; Notle SR; Meade RD; Kenny GP. 2019. Intermittent Sequential Pneumatic Compression Does Not Enhance Whole-Body Heat Loss In Elderly Adults During Extreme Heat Exposure. Appl Physiol Nutr Metab. DOI:10.1139/apnm-2019-0364

D'Souza AW; Notley SR; Kenny GP. 2020. The Relation Between Age And Sex On Whole-Body Heat Loss During Exercise-Heat Stress. Med Sci Sports Exerc. DOI:10.1249/mss.0000000000002373

Eisenman PA. 1986. Hot Weather, Exercise, Old Age, And The Kidneys. Geriatrics.

Ellis FP; Exton-Smith AN; Foster KG; Weiner JS. 1976. Eccrine Sweating And Mortality During Heat Waves In Very Young And Very Old Persons. Isr J Med Sci.

Evans E; Rendell M; Bartek J; Connor S; Bamisedun O; Dovgan D; Giitter M. 1993. Thermally-Induced Cutaneous Vasodilatation In Aging. J Gerontol. DOI:10.1093/geronj/48.2.m53

Fearnot NE; Kitoh O; Fujita T; Okamura H; Smith HJ; Calderini M. 1989. Case Studies On The Effect Of Exercise And Hot Water Submersion On Intracardiac Temperature And The Performance Of A Pacemaker Which Varies Pacing Rate Based On Temperature. Jpn Heart J. DOI:10.1536/ihj.30.353

Foster KG, Ellis FP, Dore C, Exton-Smtth AN, Weiner JS. 1976. Sweat Responses In The Aged. Age Ageing. DOI:10.1093/ageing/5.2.91

Fujii N; Dervis S; Sigal RJ; Kenny GP. 2016. Type 1 Diabetes Modulates Cyclooxygenase- And Nitric Oxide-Dependent Mechanisms Governing Sweating But Not Cutaneous Vasodilation During Exercise In The Heat. Am J Physiol Regul Integr Comp Physiol. DOI:10.1152/ajpregu.00376.2016

Fujii N; Hatam K; McGarr GW; Meade RD; Boulay P; Nishiyasu T; Kenny GP. 2019. Exogenous Activation Of Protease-Activated Receptor 2 Attenuates Cutaneous Vasodilatation And Sweating In Older Men Exercising In The Heat. Skin Pharmacol Physiol. DOI:10.1159/000500643

Fujii N; McGarr GW; Hatam K; Chandran N; Muia CM; Nishiyasu T; Boulay P; Ghassa R; Kenny GP. 2019. Heat Shock Protein 90 Does Not Contribute To Cutaneous Vasodilatation In Older Adults During Heat Stress. Microcirculation. DOI:10.1111/micc.12541

Fujii N; McGarr GW; Notley SR; Boulay P; Sigal RJ; Amano T; Nishiyasu T; Poirier MP; Kenny GP. 2021. Effects Of Short-Term Heat Acclimation On Whole-Body Heat Exchange And Local Nitric Oxide Synthase- And Cyclooxygenase-Dependent Heat Loss Responses In Exercising Older Men. Exp Physiol. DOI:10.1113/EP089025

Gagnon D; Crandall CG. 2017. Electric Fan Use During Heat Waves: Turn Off For The Elderly?. Temperature. DOI:10.1080/23328940.2017.1295833

Gagnon D; Romero SA; Cramer MN; Jay O; Crandall CG. 2016. Cardiac And Thermal Strain Of Elderly Adults Exposed To Extreme Heat And Humidity With And Without Electric Fan Use. Jama. DOI:10.1001/jama.2016.10550

Gagnon D; Romero SA; Cramer MN; Kouda K; Poh PYS; Ngo H; Jay O; Crandall CG. 2017. Age Modulates Physiological Responses During Fan Use Under Extreme Heat And Humidity. Med Sci Sports Exerc. DOI:10.1249/MSS.0000000000001348

Galetta F; Franzoni F; Femia F; Roccella N; Pentimone F; Santoro G. 2005. Lifelong Physical Training Prevents The Age-Related Impairment Of Heart Rate Variability And Exercise Capacity In Elderly People. J Sports Med Phys Fit.

Gass EM; Gass GC. 2001. Thermoregulatory Responses To Repeated Warm Water Immersion In Subjects Who Are Paraplegic. Spinal Cord. DOI:10.1038/sj.sc.3101117

Gelber DA; Pfeifer MA; Broadstone VL; Munster EW; Peterson MJ; Arezzo JC; Shamoon H; Zeidler A; Clements RS; Greene DA; Porte D; Laudadio C; Bril V. 1995. Components Of Variance For Vibratory And Thermal Threshold Testing In Normal And Diabetic Subjects. J Diabetes Complicat. DOI:10.1016/1056-8727(94)00042-m

Gerrett N; Alkemade P; Daanen H. 2021. Heat Reacclimation Using Exercise Or Hot Water Immersion. Med Sci Sports Exerc. DOI:10.1249/MSS.0000000000002612

Gerrett N; Amano T; Inoue Y; Kondo N. 2021. The Sweat Glands' Maximum Ion Reabsorption Rates Following Heat Acclimation In Healthy Older Adults. Exp Physiol. DOI:10.1113/EP088486

Gharibi V; Khanjani N; Heidari H; Ebrahimi MH; Hosseinabadi MB. 2020. The Effect Of Heat Stress On Hematological Parameters And Oxidative Stress Among Bakery Workers. Toxicol Ind Health. DOI:10.1177/0748233719899824

Greenstein D; Gupta NK; Martin P; Walker DR; Kester RC. 1995. Impaired Thermoregulation In Raynaud'S Phenomenon. Angiology. DOI:10.1177/000331979504600707

Griggs KE; Havenith G; Price MJ; Goosey-Tolfrey VL. 2019. Evaporative Heat Loss Insufficient To Attain Heat Balance At Rest In Individuals With A Spinal Cord Injury At High Ambient Temperature. J Appl Physiol. DOI:10.1152/japplphysiol.00893.2018

Guzman-Echavarria G; Middel A; Vanos J. 2022. Beyond Heat Exposure: New Methods To Quantify And Link Personal Heat Exposure, Stress, And Strain In Diverse Populations And Climates: The Journal Temperature Toolbox. Temperature. DOI:10.1080/23328940.2022.2149024

Handrakis JP; Ni Guan Z; Nulty JW; Tascione O; Rosado-Rivera D; White D; Bang C; Spungen AM; Bauman WA. 2017. Effect Of Heat Exposure On Cognition In Persons With Tetraplegia. J Neurotrauma. DOI:10.1089/neu.2016.4850

Havenith G; Inoue Y; Luttikholt V; Kenney WL. 1995. Age Predicts Cardiovascular, But Not Thermoregulatory, Responses To Humid Heat Stress. Eur J Appl Physiol Occup Physiol. DOI:10.1007/BF00601814

Hellon RF; Lind AR. 1956. Observations On The Activity Of Sweat Glands With Special Reference To The Influence Of Ageing. J Physiol. DOI:10.1113/jphysiol.1956.sp005571

Hellon RF; Lind AR. 1958. The Influence Of Age On Peripheral Vasodilatation In A Hot Environment. J Physiol. DOI:10.1113/jphysiol.1958.sp005971

Hellon RF; Lind AR; Weiner JS. 1956. The Physiological Reactions Of Men Of Two Age Groups To A Hot Environment. J Physiol. DOI:10.1113/jphysiol.1956.sp005570

Hemingway HW; Richey RE; Moore AM; Olivencia-Yurvati AH; Kline GP; Romero SA. 2022. Acute Heat Exposure Protects Against Endothelial Ischemia-Reperfusion Injury In Aged Humans. Am J Physiol Regul Integr Comp Physiol. DOI:10.1152/ajpregu.00336.2021

Holowatz AL. 2002. Age And Nitric Oxide In Cutaneous Active Vasodilation During Whole Body Heating. Thesis.

Hori S; Suzuki M; Ueno K; Sato Y; Kurihara T. 2013. Accidents During Bathing. Nihon Rinsho.

Inoue Y. 1996. Longitudinal Effects Of Age On Heat-Activated Sweat Gland Density And Output In Healthy Active Older Men. Eur J Appl Physiol Occup Physiol. DOI:10.1007/BF00376497

Inoue Y; Gerrett N; Ichinose-Kuwahara T; Umino Y; Kiuchi S; Amano T; Ueda H; Havenith G; Kondo N. 2016. Sex Differences In Age-Related Changes On Peripheral Warm And Cold Innocuous Thermal Sensitivity. Physiol Behav. DOI:10.1016/j.physbeh.2016.05.045

Jay O; Cramer MN; Ravanelli NM; Hodder SG. 2015. Should Electric Fans Be Used During A Heat Wave?. Appl Ergon. DOI:10.1016/j.apergo.2014.07.013

Jehn M; Gebhardt A; Liebers U; Kiran B; Scherer D; Endlicher W; Witt C. 2014. Heat Stress Is Associated With Reduced Health Status In Pulmonary Arterial Hypertension: A Prospective Study Cohort. Lung. DOI:10.1007/s00408-014-9587-4

Jp C; Re M. 1974. Proceedings: Physiological And Behavioural Responses Of Aged Men To Passive Heating. J Physiol.

Juzwiak I; Lewandowski Z; Kaczmarska Z; Kwass S; Markiewicz B; Pelc W; Suchowiak J; Paslawska A. 1978. Health Conditions, Heart Rate And Body Temperature In Women Working In Hot Microclimate. Pol Tyg Lek.

Kaderavek F. 1972. Thermoregulatory Adaptation To Sudden Thermal Changes In The Environment. Fysiatr Revmatol Vestn.

Kakamu T; Endo S; Hidaka T; Masuishi Y; Kasuga H; Fukushima T. 2021. Heat-Related Illness Risk And Associated Personal And Environmental Factors Of Construction Workers During Work In Summer. Sci Rep. DOI:10.1038/s41598-020-79876-w

Kanwal S; Sajid S; Nasir N; Ahsan S; Almas A. 2020. Patient-Related Factors Associated With Severe Heat-Related Illnesses In Karachi: A Hospital Perspective. J Pak Med Assoc. DOI:10.47391/JPMA.10-1016

Katschinski DM; Benndorf R; Wiedemann GJ; Mulkerin DL; Touhidi R; Robins HI. 1999. Heat Shock Protein Antibodies In Sarcoma Patients Undergoing 418 Degrees C Whole Body Hyperthermia. J Immunother. DOI:10.1097/00002371-199901000-00009

Kazman JB; Attipoe S; Kupchak BR; Deuster PA. 2020. Caffeine And Heat Have Additive But Not Interactive Effects On Physiologic Strain: A Factorial Experiment. J Therm Biol. DOI:10.1016/j.jtherbio.2020.102563

Keast ML; Adamo KB. 2000. The Finnish Sauna Bath And Its Use In Patients With Cardiovascular Disease. J Cardiopulm Rehabil. DOI:10.1097/00008483-200007000-00002

Kenney WL; Fowler SR. 1988. Methylcholine-Activated Eccrine Sweat Gland Density And Output As A Function Of Age. J Appl Physiol. DOI:10.1152/jappl.1988.65.3.1082

Kenney WL; Kamon E. 1984. Comparative Physiological Responses Of Normotensive And Essentially Hypertensive Men To Exercise In The Heat. Eur J Appl Physiol Occup Physiol. DOI:10.1007/BF00433392

Kenshalo DR. 1986. Somesthetic Sensitivity In Young And Elderly Humans. J Gerontol. DOI:10.1093/geronj/41.6.732

Kim SH; Jo SN; Myung HN; Jang JY. 2014. The Effect Of Pre-Existing Medical Conditions On Heat Stroke During Hot Weather In South Korea. Environ Res. DOI:10.1016/j.envres.2014.06.003

Kirby NV; Meade RD; Poirier MP; Sigal RJ; Boulay P; Kenny GP. 2023. Association Between Haemoglobin A1C And Whole-Body Heat Loss During Exercise-Heat Stress In Physically Active Men With Type 2 Diabetes. Exp Physiol. DOI:10.1113/EP090915

Knapik Z; Lubczynska-Kowalska W; Koziorowski C. 1987. Changes In Internal Body Temperature After Physical Exertion Under Heat Discomfort Conditions As A Parameter Of Biological Age. Pol Tyg Lek.

Kovalenko VP; Sulimo-Samuillo EK. 1983. Physiological Reactions To Repeated Application Of High Temperature In Persons Of Various Age Groups. Voen Med Zh.

Kuhlemeier KV; Miller JM. 1978. Pulse Rate-Rectal Temperature Relationships During Prolonged Work. J Appl Physiol Respir Environ Exerc Physiol. DOI:10.1152/jappl.1978.44.3.450

Kuhlemeier KV; Miller JM; Dukes-Dobos FN; Jensen R. 1977. Determinants Of The Prescriptive Zone Of Industrial Workers. J Appl Physiol Respir Environ Exerc Physiol. DOI:10.1152/jappl.1977.43.2.347

Kumar NS; Bart J; Barton C; Graham ML; Leung PP; Tittley TD; Lee I; Bang C; Bauman WA; Handrakis JP. 2021. Core Temperature Lability Predicts Sympathetic Interruption And Cognitive Performance During Heat Exposure In Persons With Spinal Cord Injuries. J Neurotrauma. DOI:10.1089/neu.2020.7598

Kupchak BR; Kazman JB; Vingren JL; Levitt DE; Lee EC; Williamson KH; Armstrong LE; Deuster PA. 2017. Blood Hemostatic Changes During An Ultraendurance Road Cycling Event In A Hot Environment. Wilderness Environ Med. DOI:10.1016/j.wem.2017.05.002

Lamarche DT; Meade RD; D'Souza AW; Flouris AD; Hardcastle SG; Sigal RJ; Boulay P; Kenny GP. 2017. The Recommended Threshold Limit Values For Heat Exposure Fail To Maintain Body Core Temperature Within Safe Limits In Older Working Adults. J Occup Environ Hyg. DOI:10.1080/15459624.2017.1321844

Laouadi A; Ji L; Shu C; Wang L; Lacasse MA. 2023. Overheating Risk Analysis In Long-Term Care Homes: Development Of Overheating Limit Criteria. Buildings. DOI:10.3390/buildings13020390

Leppaluoto J; Tapanainen P; Knip M. 1987. Heat Exposure Elevates Plasma Immunoreactive Growth Hormone-Releasing Hormone Levels In Man. J Clin Endocrinol Metab. DOI:10.1210/jcem-65-5-1035

Lima MV; Ochiai ME; Vieira KN; Scipioni A; Cardoso JN; Munhoz RT; Morgado PC; Barretto AC. 2014. Thermal Vasodilation Using A Portable Infrared Thermal Blanket In Decompensated Heart Failure. Int Heart J. DOI:10.1536/ihj.14-096

Lind AR; Humphreys PW; Collins KJ; Foster K; Sweetland KF. 1970. Influence Of Age And Daily Duration Of Exposure On Responses Of Men To Work In Heat. J Appl Physiol. DOI:10.1152/jappl.1970.28.1.50

Lindemann U; Skelton DA; Oksa J; Beyer N; Rapp K; Becker C; Klenk J. 2018. Social Participation And Heat-Related Behavior In Older Adults During Heat Waves And On Other Days. Z Gerontol Geriatr. DOI:10.1007/s00391-017-1338-8

Lindemann U; Stotz A; Beyer N; Juha O; Skelton DA; Becker C; Rapp K; Klenk J. 2017. Effect Of Indoor Temperature On Physical Performance In Older Adults During Days With Normal Temperature And Heat Waves. Int J Environ Res Public Health. DOI:10.3390/ijerph14020186

Ljungberg AS; Enander A; Holmer I. 1979. Evaluation Of Heat Stress During Sedentary Work. Scand J Work Environ Health. DOI:10.5271/sjweh.2669

Mac VV; Elon L; Smith DJ; Tovar-Aguilar A; Economos E; Flocks J; Hertzberg V; McCauley L. 2021. A Modified Physiological Strain Index For Workplace-Based Assessment Of Heat Strain Experienced By Agricultural Workers. Am J Ind Med. DOI:10.1002/ajim.23230

Macartney MJ; Notley SR; Meade RD; Herry CL; Kenny GP. 2020. Heart Rate Variability In Older Men On The Day Following Prolonged Work In The Heat. J Occup Environ Hyg. DOI:10.1080/15459624.2020.1779932

Mackinnon Pamela CB. 1954. Variations With Age In The Number Of Active Palmar Digital Sweat Glands. J Neurol Neurosurg Psychiatry.

McCormick JJ; King KE; Cote MD; Meade RD; Akerman AP; Kenny GP. 2021. Impaired Autophagy Following Ex Vivo Heating At Physiologically Relevant Temperatures In Peripheral Blood Mononuclear Cells From Elderly Adults. J Therm Biol. DOI:10.1016/j.jtherbio.2020.102790

McGinn R; Poirier MP; Louie JC; Sigal RJ; Boulay P; Flouris AD; Kenny GP. 2017. Increasing Age Is A Major Risk Factor For Susceptibility To Heat Stress During Physical Activity. Appl Physiol Nutr Metab. DOI:10.1139/apnm-2017-0322

Meade RD; Akerman AP; Notley SR; Kirby NV; Kenny P. 2022. Body Temperature And Cardiovascular Regulation In Older Adults During 8-Hour Exposures To Ambient Conditions Experienced Indoors During Extreme Heat Events: A Randomized Crossover Trial. FASEB J.

Meade RD; Lauzon M; Poirier MP; Flouris AD; Kenny GP. 2015. An Evaluation Of The Physiological Strain Experienced By Electrical Utility Workers In North America. J Occup Environ Hyg. DOI:10.1080/15459624.2015.1043054

Mitchell DC; Castro J; Armitage TL; Vega-Arroyo AJ; Moyce SC; Tancredi DJ; Bennett DH; Jones JH; Kjellstrom T; Schenker MB. 2017. Recruitment, Methods, And Descriptive Results Of A Physiologic Assessment Of Latino Farmworkers: The California Heat Illness Prevention Study. J Occup Environ Med. DOI:10.1097/JOM.0000000000000988

Morgan CL; Nadas AS. 1963. Sweating And Congestive Heart Failure. N Engl J Med. DOI:10.1056/nejm196303142681104

Morley J; Beauchamp G; Suyama J; Guyette FX; Reis SE; Callaway CW; Hostler D. 2012. Cognitive Function Following Treadmill Exercise In Thermal Protective Clothing. Eur J Appl Physiol. DOI:10.1007/s00421-011-2144-4

Morris NB; Chaseling GK; English T; Gruss F; Maideen MFB; Capon A; Jay O. 2021. Electric Fan Use For Cooling During Hot Weather: A Biophysical Modelling Study. Lancet Planet Health. DOI:10.1016/S2542-5196(21)00136-4

Moyce S; Armitage T; Mitchell D; Schenker M. 2020. Acute Kidney Injury And Workload In A Sample Of California Agricultural Workers. Am J Ind Med. DOI:10.1002/ajim.23076

Moyce S; Mitchell D; Armitage T; Tancredi D; Joseph J; Schenker M. 2017. Heat Strain, Volume Depletion And Kidney Function In California Agricultural Workers. Occup Environ Med. DOI:10.1136/oemed-2016-103848

Mukhopadhyay B; Weitz CA. 2022. Heat Exposure, Heat-Related Symptoms And Coping Strategies Among Elderly Residents Of Urban Slums And Rural Vilages In West Bengal, India. Int J Environ Res Public Health. DOI:10.3390/ijerph191912446

Notley SR; Meade RD; D'Souza AW; Friesen BJ; Kenny GP. 2018. Heat Loss Is Impaired In Older Men On The Day After Prolonged Work In The Heat. Med Sci Sports Exerc. DOI:10.1249/MSS.0000000000001643

Notley SR; Meade RD; D'Souza AW; McGarr GW; Kenny GP. 2018. Cumulative Effects Of Successive Workdays In The Heat On Thermoregulatory Function In The Aging Worker. Temperature. DOI:10.1080/23328940.2018.1512830

Ohara K; Sato H; Takaba S. 1974. Correlative Relationships Of Response Patterns Between Body Temperature, Sweat Rate And Sodium Concentration In Sweat During Heat Exposure In Man. Jpn J Physiol. DOI:10.2170/jjphysiol.24.19

Okamoto-Mizuno K; Tsuzuki K; Mizuno K. 2004. Effects Of Mild Heat Exposure On Sleep Stages And Body Temperature In Older Men. Int J Biometeorol. DOI:10.1007/s00484-004-0209-3

O'lenick CR; Baniassadi A; Michael R; Monaghan A; Boehnert J; Yu X; Hayden MH; Wiedinmyer C; Zhang K; Crank PJ; Heusinger J; Hoel P; Sailor DJ; Wilhelmi OV. 2020. A Case-Crossover Analysis Of Indoor Heat Exposure On Mortality And Hospitalizations Among The Elderly In Houston, Texas. Environ Health Perspect. DOI:10.1289/EHP6340

Ou Y; Wang F; Zhao J; Deng Q. 2023. Risk Of Heatstroke In Healthy Elderly During Heatwaves: A Thermoregulatory Modeling Study. Build Environ. DOI:10.1016/j.buildenv.2023.110324

Parikh DJ; Ghodasara NB; Ramanathan NL. 1978. A Special Thermal Stress Problem In Ceramic Industry. Eur J Appl Physiol Occup Physiol. DOI:10.1007/BF00420990

Peftiev IF; Maksimovich VA. 1989. Signs Of Chronic Overheating In Miners Of Deep Coal Mines. Gig Tr Prof Zabol.

Peiffer JJ; Abbiss CR. 2013. Thermal Stress In North Western Australian Iron Ore Mining Staff. Ann Occup Hyg. DOI:10.1093/annhyg/mes084

Petrofsky J; Lee H; Trivedi M; Hudlikar AN; Yang CH; Goraksh N; Alshammari F; Mohanan M; Soni J; Agilan B; Pai N; Chindam T; Murugesan V; Yim JE; Katrak V. 2010. The Influence Of Aging And Diabetes On Heat Transfer Characteristics Of The Skin To A Rapidly Applied Heat Source. Diabetes Technol Ther. DOI:10.1089/dia.2010.0152

Petrofsky JS; Besonis C; Rivera D; Schwab E; Lee S. 2003. Heat Tolerance In Patients With Type Ii Diabetes. J Appl Res.

Petrofsky JS; Lee S; Patterson C; Cole M; Stewart B. 2005. Sweat Production During Global Heating And During Isometric Exercise In People With Diabetes. Med Sci Monit.

Petrofsky JS; McLellan K; Bains GS; Prowse M; Ethiraju G; Lee S; Gunda S; Lohman E; Schwab E. 2008. Skin Heat Dissipation: The Influence Of Diabetes, Skin Thickness, And Subcutaneous Fat Thickness. Diabetes Technol Ther. DOI:10.1089/dia.2008.0009

Phillips PA; Bretherton M; Johnston CI; Gray LC. 1991. Reduced Osmotic Thirst In Healthy Elderly Men. Am J Physiol. DOI:10.1152/ajpregu.1991.261.1.r166

Pirnay F; Petit JM; Deroanne R. 1969. Comparative Evolution Of Heart Rate And Body Temperature During Muscular Exercise At High Temperature. Int Z Angew Physiol.

Proano E; Perbeck L. 1994. Effect Of Exposure To Heat And Intake Of Ethanol On The Skin Circulation And Temperature In Ischaemic Limbs. Clin Physiol. DOI:10.1111/j.1475-097x.1994.tb00387.x

Provins KA; Bell CR. 1970. Effects Of Heat Stress On The Performance Of Two Tasks Running Concurrently. J Exp Psychol. DOI:10.1037/h0029557

Puchalska H; Kozlowski S. 1969. Relation Of Physiological Loading In Female Workers In High Temperature To Their Age. Acta Physiol Pol.

Raven PB; Drinkwater BL; Horvath SM; Ruhling RO; Gliner JA; Sutton JC; Bolduan NW. 1974. Age, Smoking Habits, Heat Stress, And Their Interactive Effects With Carbon Monoxide And Peroxyacetylnitrate On Man'S Aerobic Power. Int J Biometeorol. DOI:10.1007/BF01453936

Raynaud J; Martineaud JP; Bhatnagar OP; Viellefond H; Durand J. 1976. Body Temperatures During Rest And Exercise In Residents And Sojourners In Hot Climate. Int J Biometeorol. DOI:10.1007/BF01553588

Razmjou S; Kjellberg A. 1992. Sustained Attention And Serial Responding In Heat: Mental Effort In The Control Of Performance. Aviat Space Environ Med.

Romberg A; Ikonen A; Ruutiainen J; Virtanen A; Hamalainen P. 2012. The Effects Of Heat Stress On Physical Functioning In Persons With Multiple Sclerosis. J Neurol Sci. DOI:10.1016/j.jns.2012.05.024

Ruiperez Cantera I; Sepulveda Moya D. 2004. Temperature And Death In Old People. Med Clin. DOI:10.1016/s0025-7753(04)74398-5

Saari A; Tolonen U; Paakko E; Suominen K; Jauhiainen J; Sotaniemi KA; Myllyla VV. 2009. Sweating Impairment In Patients With Multiple Sclerosis. Acta Neurol Scand. DOI:10.1111/j.1600-0404.2009.01164.x

Saito H; Kogure K. 1989. Thermal Sudomotor Deficits In Parkinson'S Disease. Rinsho Shinkeigaku.

Schlader ZJ; Ganio MS; Pearson J; Lucas RA; Gagnon D; Rivas E; Kowalske KJ; Crandall CG. 2015. Heat Acclimation Improves Heat Exercise Tolerance And Heat Dissipation In Individuals With Extensive Skin Grafts. J Appl Physiol. DOI:10.1152/japplphysiol.00176.2015

Senay LC Jr. 1972. Changes In Plasma Volume And Protein Content During Exposures Of Working Men To Various Temperatures Before And After Acclimatization To Heat: Separation Of The Roles Of Cutaneous And Skeletal Muscle Circulation. J Physiol. DOI:10.1113/jphysiol.1972.sp009881

Shin TW; Wilson M; Wilson TW. 2003. Are Hot Tubs Safe For People With Treated Hypertension?. Cmaj.

Shishliannikova GI; Dement'eva LN; Popova NA. 1983. Functional State Of The Body And Various Specific Functions In Women Engaged In Construction Work In Hot Climate. Gig Tr Prof Zabol.

Shleifman FM; Kucheruk TK. 1985. Mechanism Of Thermoregulation And Immunological Reactions In Women Working In Hot Microclimate. Vrach Delo.

Shleifman FM; Tashker ID; Lashchuk AA; Vialaia LS; Shakhbazian EV. 1990. Body Function And The Biological Aging Of Those Working Under The Conditions Of A Warming Microclimate. Vrach Delo.

Shleifman FM; Tashker ID; Lashchuk AA; Vialaia LS; Shakhbazian EV. 1990. Results Of Studies On Long-Term Results Of The Effects Of Hot Microclimate On The Body Of Workers. Gig Tr Prof Zabol.

Solonin IuG; Katsiuba EA. 2003. Thermoregulation And Blood Circulation In Adults During Short Term Exposure To Extreme Temperatures. Fiziol Cheloveka. DOI:10.1023/A:1022950728495

Spector JT; Bonauto DK; Sheppard L; Busch-Isaksen T; Calkins M; Adams D; Lieblich M; Fenske RA. 2016. A Case-Crossover Study Of Heat Exposure And Injury Risk In Outdoor Agricultural Workers. Plos One. DOI:10.1371/journal.pone.0164498

Spioch FM. 1979. Effect Of Long-Term Work In Hot Microclimate On Various Physiological Parameters In Metallurgists. Med Pr.

Stephan F; Ghiglione S; Decailliot F; Yakhou L; Duvaldestin P; Legrand P. 2005. Effect Of Excessive Environmental Heat On Core Temperature In Critically Ill Patients An Observational Study During The 2003 European Heat Wave. Br J Anaesth. DOI:10.1093/bja/aeh291

Sturdee DW; Wilson KA; Pipili E; Crocker AD. 1978. Physiological Aspects Of Menopausal Hot Flush. Br Med J. DOI:10.1136/bmj.2.6130.79

Tabassum Shaesta; Raza Naila; Shah Syed Zubair. 2019. Outcome Of Heat Stroke Patients Referred To A Tertiary Hospital In Pakistan: A Retrospective Study. Emhj. DOI:10.26719/emhj.18.059

Taylor NA; Allsopp NK; Parkes DG. 1995. Preferred Room Temperature Of Young Vs Aged Males: The Influence Of Thermal Sensation, Thermal Comfort, And Affect. J Gerontol A Biol Sci Med Sci. DOI:10.1093/gerona/50a.4.m216

Tei C; Horikiri Y; Park JC; Jeong JW; Chang KS; Toyama Y; Tanaka N. 1995. Acute Hemodynamic Improvement By Thermal Vasodilation In Congestive Heart Failure. Circulation. DOI:10.1161/01.cir.91.10.2582

Tew GA; Saxton JM; Klonizakis M; Moss J; Ruddock AD; Hodges GJ. 2011. Aging And Aerobic Fitness Affect The Contribution Of Noradrenergic Sympathetic Nerves To The Rapid Cutaneous Vasodilator Response To Local Heating. J Appl Physiol. DOI:10.1152/japplphysiol.01423.2010

Teyton A; Tremblay M; Tardif I; Lemieux MA; Nour K; Benmarhnia T. 2022. A Longitudinal Study On The Impact Of Indoor Temperature On Heat-Related Symptoms In Older Adults Living In Non-Air-Conditioned Households. Ehp. DOI:10.1289/EHP10291

Thomas KN; van Rij AM; Lucas SJE; Cotter JD. 2017. Lower-Limb Hot-Water Immersion Acutely Induces Beneficial Hemodynamic And Cardiovascular Responses In Peripheral Arterial Disease And Healthy, Elderly Controls. Am J Physiol Regul Integr Comp Physiol. DOI:10.1152/ajpregu.00404.2016

Tokizawa K; Sawada S; Tai T; Lu J; Oka T; Yasuda A; Takahashi M. 2015. Effects Of Partial Sleep Restriction And Subsequent Daytime Napping On Prolonged Exertional Heat Strain. Occup Environ Med. DOI:10.1136/oemed-2014-102548

Trbovich MB; Kiratli JB; Price MJ. 2016. The Effects Of A Heat Acclimation Protocol In Persons With Spinal Cord Injury. J Therm Biol. DOI:10.1016/j.jtherbio.2016.10.006

Trezza BM; Apolinario D; de Oliveira RS; Busse AL; Goncalves FL; Saldiva PH; Jacob-Filho W. 2015. Environmental Heat Exposure And Cognitive Performance In Older Adults: A Controlled Trial. Age. DOI:10.1007/s11357-015-9783-z

Waldock KAM; Hayes M; Watt PW; Maxwell NS. 2018. Physiological And Perceptual Responses In The Elderly To Simulated Daily Living Activities In Uk Summer Climatic Conditions. Public Health. DOI:10.1016/j.puhe.2018.04.012

Walker A; Beatty HEW; Zanetti S; Rattray B. 2017. Improving Body Composition May Reduce The Immune And Inflammatory Responses Of Firefighters Working In The Heat. J Occup Environ Med. DOI:10.1097/JOM.0000000000000980

Wang FM; Deng QH; Lei TH; Wang XM; Wang AR. 2023. Biophysical Modelling Predicts Unreliable Core Temperature Responses On Healthy Older Adults Using Electric Fans At Residential Homes During Heatwaves. Build Environ. DOI:10.1016/j.buildenv.2022.109888

Weems C; Olson W; Nichols GR 2nd. 1985. Risk Factors For Death During A Heat Wave. J Ky Med Assoc.

Wong del PL; Chung JW; Chan AP; Wong FK; Yi W. 2014. Comparing The Physiological And Perceptual Responses Of Construction Workers (Bar Benders And Bar Fixers) In A Hot Environment. Appl Ergon. DOI:10.1016/j.apergo.2014.06.002

Wright HE; Larose J; McLellan TM; Miller S; Boulay P; Kenny GP. 2013. Do Older Firefighters Show Long-Term Adaptations To Work In The Heat?. J Occup Environ Hyg. DOI:10.1080/15459624.2013.821574

Yamasaki M; Kim KT; Choi SW; Muraki S; Shiokawa M; Kurokawa T. 2001. Characteristics Of Body Heat Balance Of Paraplegics During Exercise In A Hot Environment. J Physiol Anthropol Appl Human Sci. DOI:10.2114/jpa.20.227

Yamasaki M; Shiokawa M; Choi SW; Muraki S. 2000. Effect Of Acute Heat Exposure On Skin Blood Flow Of The Paralyzed Thigh In Persons With Spinal Cord Injury. Spinal Cord. DOI:10.1038/sj.sc.3100988

Yau AM; Moss AD; James LJ; Gilmore W; Ashworth JJ; Evans GH. 2015. The Influence Of Angiotensin Converting Enzyme And Bradykinin Receptor B2 Gene Variants On Voluntary Fluid Intake And Fluid Balance In Healthy Men During Moderate-Intensity Exercise In The Heat. Appl Physiol Nutr Metab. DOI:10.1139/apnm-2014-0307

Yousef MK; Dill DB; Vitez TS; Hillyard SD; Goldman AS. 1984. Thermoregulatory Responses To Desert Heat: Age, Race And Sex. J Gerontol. DOI:10.1093/geronj/39.4.406

Yousef MK; Sagawa S; Shiraki K. 1986. Thermoregulatory Responses Of The Elderly Population. J UOEH. DOI:10.7888/juoeh.8.219

Zajdlikova E; Kolesar J; Ferencikova J. 1982. Changes In The Coagulation And Fibrinolytic System After Hyperthermia In Healthy And Ill Subjects. Fysiatr Revmatol Vestn.
